# Chromogranin A and its fragments in cardiovascular, immunometabolic, and cancer regulation

**DOI:** 10.1111/nyas.14249

**Published:** 2019-10-06

**Authors:** Sushil K. Mahata, Angelo Corti

**Affiliations:** ^1^ VA San Diego Healthcare System San Diego California; ^2^ Metabolic Physiology & Ultrastructural Biology Laboratory, Department of Medicine University of California San Diego La Jolla California; ^3^ IRCCS San Raffaele Scientific Institute San Raffaele Vita‐Salute University Milan Italy

**Keywords:** pancreastatin, vasostatin, catestatin, cardiovascular diseases, immunometabolism, cancer

## Abstract

Chromogranin A (CgA)—the index member of the chromogranin/secretogranin secretory protein family—is ubiquitously distributed in endocrine, neuroendocrine, and immune cells. Elevated levels of CgA‐related polypeptides, consisting of full‐length molecules and fragments, are detected in the blood of patients suffering from neuroendocrine tumors, heart failure, renal failure, hypertension, rheumatoid arthritis, and inflammatory bowel disease. Full‐length CgA and various CgA‐derived peptides, including vasostatin‐1, pancreastatin, catestatin, and serpinin, are expressed at different relative levels in normal and pathological conditions and exert diverse, and sometime opposite, biological functions. For example, CgA is overexpressed in genetic hypertension, whereas catestatin is diminished. In rodents, the administration of catestatin decreases hypertension, cardiac contractility, obesity, atherosclerosis, and inflammation, and it improves insulin sensitivity. By contrast, pancreastatin is elevated in diabetic patients, and the administration of this peptide to obese mice decreases insulin sensitivity and increases inflammation. CgA and the N‐terminal fragment of vasostatin‐1 can enhance the endothelial barrier function, exert antiangiogenic effects, and inhibit tumor growth in animal models, whereas CgA fragments lacking the CgA C‐terminal region promote angiogenesis and tumor growth. Overall, the CgA system, consisting of full‐length CgA and its fragments, is emerging as an important and complex player in cardiovascular, immunometabolic, and cancer regulation.

## Graphical abstract

In this review, we focus on how full‐length chromogranin A and its peptides, including PST, vasostatin‐1, CST, and serpinin, as important modulators of cardiovascular functions, immunometabolism, and cancer.

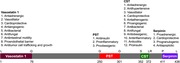

## Introduction

**Figure 1:**
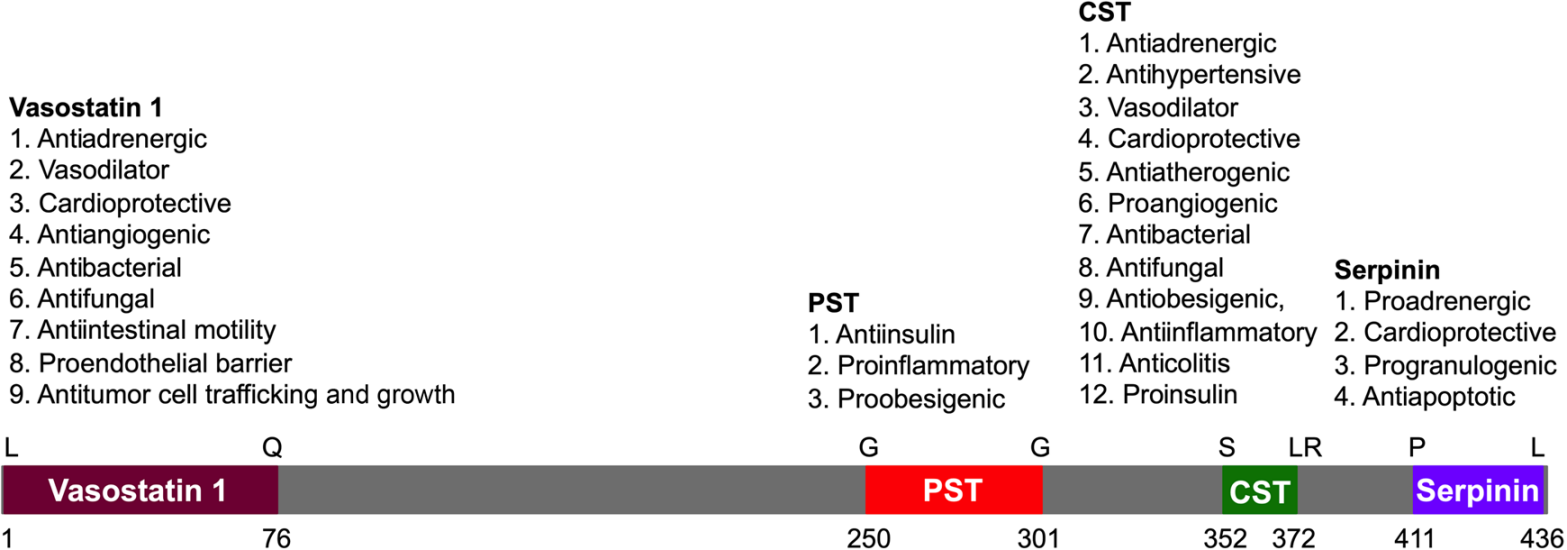
Schematic diagram showing the major peptide domains of CgA protein. Relative locations of vasostatin‐1, pancreastatin (PST), catestatin (CST), and serpinin domains along with the first and last amino acids for each peptide domain are shown except for CST, where an arginine at position 373 is shown in addition to the last amino acid. Major functions of each of the peptide are shown above the peptide domain.

In September 1965, Banks and Helle reported the release of proteins from the stimulated adrenal medulla.^1^ In July 1967, Blaschko's group named splanchnic nerve–stimulated release of soluble proteins “chromogranins;”^2^ and in September 1967, his group coined the term *chromogranin A* (CgA) for the major component of these proteins.^3^ Subsequently, it has been reported that patients suffering from neuroendocrine tumors, heart failure (HF), renal failure, hypertension, rheumatoid arthritis, and inflammatory bowel disease display elevated levels of circulating CgA‐related polypeptides consisting of full‐length molecules and fragments (Table 1). The human chromogranin A gene (*CHGA*; protein abbreviation hCgA) encodes a 439‐amino‐acid mature protein of approximately 48–52 kDa with a coiled‐coil structure.^4^, ^5^, ^6^, ^7^, ^8^ Structurally, hCgA has nine dibasic sites and is proteolytically cleaved by prohormone convertases,^9^, ^10^, ^11^ cathepsin L,^12^ plasmin,^13^, ^14^, ^15^ and kallikrein^16^ to generate biologically active peptides, including (1) the dysglycemic peptide pancreastatin (PST: hCgA_250–301_);^17^, ^18^ (2) WE14 (hCgA_324–337_), which acts as the antigen for highly diabetogenic CD4^+^ T cell clones;^19^, ^20^, ^21^ (3) the vasodilating, antiadrenergic, and antiangiogenic peptide vasostatin‐1 (hCgA_1–76_);^22^, ^23^, ^24^, ^25^, ^26^ (4) the antiadrenergic, antihypertensive, antibacterial, proangiogenic, antiobesogenic, and antiinflammatory peptide catestatin (CST: hCgA_352–372_);^27^, ^28^, ^29^, ^30^, ^31^, ^32^, ^33^, ^34^, ^35^, ^36^, ^37^, ^38^, ^39^, ^40^, ^41^ and (5) the proadrenergic peptide serpinin (hCgA_411–436_).^42^, ^43^ Several of these CgA‐derived peptides exhibit opposing (i.e., with regard to each other) regulatory effects. For example, cardiac contractility in rodents is controlled by vasostatin‐1 and CST, which are antiadrenergic,^36^, ^44^ as well as by serpinin, which is proadrenergic.^43^ Likewise, angiogenesis is controlled by vasostatin‐1 functioning in an antiangiogenic manner^26^, ^41^ and CST functioning in a proangiogenic manner^35^, ^41^ (Fig. 1). CgA gene (*Chga*) knockout (KO) was generated by targeted deletion of exon 1 and approximately 1.5 kbp of its proximal promoter, resulting in complete elimination of *Chga* expression. *Chga*‐KO mice provide an important model to study the roles of individual CgA‐derived peptides through analysis of phenotypes after supplementation.^33^, ^40^, ^45^, ^46^, ^47^ Here, we focus on how full‐length CgA and its peptides, including PST, vasostatin‐1, CST, and serpinin, are important modulators of cardiovascular functions, immunometabolism, and cancer.

**Table 1:** Plasma CgA levels in health and diseases

Diseases/abnormalities	Diseases (nM)	Controls (nM)	References
**Cardiovascular diseases**			
Controls		2.64	O'Connor^60^
Hypertension (primary)	4.04		
Hypertension (primary)	4.05		O'Connor^61^
Hypertension (secondary)	3.93		
Controls		3.29	Cryer^207^
Cardiac arrest	3.23		
Controls		1.46	Ceconi *et al*.^52^
CHF NYHA I	2.25		
CHF NYHA II	3		
CHF NYHA III	5.7		
CHF NYHA IV	11.4		
Controls		0.49	Omland *et al*.^53^
Myocardial infarction	0.61		
Controls		1.31	Pieroni *et al*.^48^
DCM	3.14		
HCM	3.07		
Controls		1.0	Larsen *et al*.^71^
CHF NYHA II and III	2.1		
Controls		0.98	Tombetti^208^
Takayasu arteritis	2.36		
**Renal diseases**			
Controls		0.57	O'Connor^209^
Renal failure	7.99		
Controls		1.16	Spadaro^210^
Chronic renal failure	28.41		
**Liver diseases**			
Controls		0.57	O'Connor^209^
Liver dysfunction	2.04		
Controls		1.16	Spadaro^210^
Liver cirrhosis	5.09		
**Lung diseases**			
Controls		2.51	Sobol^211^
COPD	3.45		
**Inflammatory bowel disease**			
Controls		1.16	Spadaro^210^
IBD	4.46		
**Rheumatoid arthritis**			
Controls		1.13	Di Comite^203^
Rheumatoid arthritis	5.79		
**Sepsis**			
Controls		4.86	Hsu^212^
Severe sepsis	9.89		
Controls		1.76	Zhang^213^
Severe sepsis	3.34		
Controls		<4.0	Rosjo^214^
Sepsis survivors	9.1		
Sepsis nonsurvivors	14.0		
**Meniere's disease**			
Controls		0.67	Teggi^215^
Meniere's disease	1.46		
**Erdheim–Chester disease**			
Controls		0.72	Ferrero^216^
Erdheim–Chester disease	3.13		
**Tumors/cancer**			
Controls		2.64	O'Connor^61^
Pheochromocytoma	32.99		
Controls		1.17	O'Connor^217^
Pheochromocytoma	15.04		
Carcinoid tumor	723.68		
Pancreatic islet tumor	101.39		
Thyroid carcinoma	11.53		
Parathyroid adenoma	4.46		
Controls		2.51	Sobol^211^
Small cell lung carcinoma	16.66		
Large cell lung carcinoma	3.74		
Adenocarcinoma	3.68		
Controls		0.57	Hsiao^218^
Pheochromocytoma (familial)	2.41		
Pheochromocytome (sporadic)	3.76		
Controls		3.22	Cryer^207^
Pheochromocytoma	6.81		
Controls		0.29	Blind^219^
Medullary thyroid carcinoma	16.48		
Controls		1.16	Spadaro^210^
Hepatocellular carcinoma	15.04		
Controls		1.19	Lee *et al*.^220^
Pancreatic ductal adenocarcinoma	2.87		

## Cardiovascular functions

### Circulating CgA in patients with cardiovascular diseases

Several studies have addressed the role of circulating CgA as a biomarker in patients with cardiovascular diseases using CgA‐ELISA systems that were unable to discriminate between the full‐length CgA and its fragments. Using these assays, increased levels of immunoreactive CgA–related polypeptides have been detected in the blood of patients with myocardial infarction, HF, decompensated and hypertrophic heart, and acute coronary syndromes, with important prognostic implications in several cases.^48^, ^49^, ^50^, ^51^, ^52^, ^53^, ^54^, ^55^ Studies in patients with dilated and hypertrophic cardiomyopathy revealed that plasma CgA (detected by a homemade ELISA that was unable to discriminate the full‐length CgA and its fragments) strongly correlates with circulating B‐type natriuretic peptide (BNP), an important diagnostic and prognostic marker of HF.^48^ A later study performed in patients with acute destabilized HF showed that CgA adds independent prognostic information to N‐terminal proBNP, another known prognostic marker of the disease, thus showing an improved prognostic ability of the combination of the two markers.^56^ However, the prognostic value of CgA in acute and chronic myocardial failure is still a matter of debate.^57^ Indeed, results of a trial conducted on a large group of patients with chronic HF (GISSI, *Gruppo Italiano per lo Studio della Sopravvivenza nell'Infarto Miocardico*) showed CgA association with all‐cause mortality or cardiovascular morbidity in univariate, but not multivariate, regression analysis adjusted for known risk factors.^54^ Given the highly heterogeneous nature of the CgA antigen owing to differential post‐translational modifications and proteolytic processing, and considering that CgA and its fragments can work as cardioregulatory hormones in certain cases with opposite functions (discussed below), the detection of specific CgA‐related polypeptides with specific immunoassays is necessary to fully assess the prognostic value of the CgA system in cardiovascular diseases. This concept is well exemplified by the results of a recent study showing that serum levels of the hCgA_1–113_ fragment (known as vasostatin‐2) are reduced in patients with ischemic chronic HF and are negatively, and independently, associated with major adverse cardiac effects.^58^ The role of CgA and its fragments on cardiovascular functions will be discussed below.

### CgA in hypertension

O'Connor's group was the first to report that circulating levels of CgA are associated with hypertension. In 1984, they reported a normal plasma CgA level of 2.64 nM, which was increased by ∼12‐fold (32.99 nM) in pheochromocytoma patients,^59^ as well as ∼1.5‐fold in hypertensive patients (4.04 nM).^60^ His group also reported that plasma CgA levels are increased in both essential (primary) hypertension (4.05 versus 2.64 nM) and secondary hypertension (3.93 versus 2.64 nM).^61^ Since blocking sympathetic outflow by guanabenz resulted in reduced plasma CgA, it was suggested that plasma CgA is, at least in part, regulated by sympathetic nerve, and essential hypertension could represent an increased sympathoadrenal activity.^61^ Subsequently, increased CgA content was reported both in the adrenal gland^62^, ^63^ and plasma.^63^

### CgA in HF

Characterized by defective ventricular filling or the ejection of blood, HF is caused by structural and functional defects in myocardium. The major pathogenic mechanisms involved in HF include increased hemodynamic overload, ischemia‐associated dysfunction, ventricular remodeling, abnormal myocyte calcium handling, and excessive neurohumoral stimulation.^64^ Longstanding hypertension is one of the major causes of HF; and most patients with HF show a history of hypertension.^65^, ^66^ Augmented sympathetic nerve activity is also recognized as a major contributor to the complex pathophysiology of HF.^67^, ^68^ Since sympathoexcitation has been reported to increase plasma CgA, studies were undertaken to evaluate the possible association of CgA with HF.^61^, ^69^ A study was conducted with 103 healthy subjects and 160 consecutive elective patients (83.1% men; 16.9% women) with chronic heart failure (CHF), among which patients were classified in accordance with New York Heart Association (NYHA): NHYA I, NHYA II, NHYA III, and NHYA IV, to find out whether plasma level of CgA is increased in CHF subjects. Because of a nonnormal distribution, only median values were presented, which revealed that the increase in CgA in plasma was positively correlated with the severity of the diseases (normal subjects, 1.46 nM; NYHA I, 2.25 nM; NYHA II, 3 nM; NYHA III, 5.7 nM; and NYHA IV, 11.14 nM).^52^ Of note, although CgA did not correlate with CHF‐activated hormones, such as catecholamines, vasopressin, endothelins, and components of the renin–angiotensin–aldosterone system, it did correlate with the soluble tumor necrosis factor‐alpha (TNF‐α) receptors and, to a lower extent, with TNF‐α itself.^70^ Another study of 20 healthy volunteers and 25 men with stable CHF (NYHA II and NYHA III) also showed elevated plasma CgA (2.1 versus 1.0 nM controls);^71^ this study also confirmed a positive correlation between CgA and TNF‐α, as well as between CgA and N‐terminal atrial natriuretic peptide. Since plasma catecholamine levels were comparable, the results suggested that inflammatory responses contribute more to elevated plasma CgA in CHF than to activation of the sympathoadrenergic system.

To find out whether plasma CgA could serve as a prognostic marker for myocardial infarction, Omland's group measured plasma CgA in 119 patients (88 men; 31 women) on day 3 after the onset of myocardial infarction.^53^ They found significantly higher plasma CgA levels in patients with symptoms and signs of HF (0.61 versus 0.49 nM); the mortality rate was significantly higher in patients with plasma CgA of >0.49 nM. They concluded that plasma CgA levels are associated with long‐term mortality after myocardial infarction.^53^ In a subsequent study in 217 patients with acute myocardial infarction complicated with HF or ventricular dysfunction, plasma CgA emerged as a strong and independent predictor of death and hospitalization.^49^ In another study in 137 patients (94% men; 6% women) admitted to a single center with acute decompensated HF, CgA was found to exhibit comparable prognostic accuracy to N‐terminal probrain natriuretic peptide (NT‐proBNP) and provided further prognostic information after adjustment for other risk factors, including estimated glomerular filtration rate and NT‐proBNP.^56^

One of the largest studies evaluating the prognostic value of CgA in 1233 chronic HF patients (GISSI: 86.4% men and 13.6% women) found that plasma CgA concentrations are increased in proportion to disease severity.^54^ The estimated probability of death was calculated to be the highest with CgA concentrations >33 U/L and lowest with CgA concentrations <16 U/L. Univariate analyses found a correlation between baseline CgA levels with the two coprimary outcome measures in GISSI‐HF: mortality and mortality of hospitalization for cardiovascular disease during follow‐up. The study found significant correlations between plasma CgA levels and BNP and hs‐C‐reactive protein levels. However, multivariate analyses did not find significant association between elevated CgA levels and mortality or CgA levels and either mortality or cardiovascular morbidity during follow up. Thus, by these data, it was concluded that plasma CgA levels in patients with chronic, stable HF do not provide incremental prognostic information over that obtained from physical examination, routine biochemical analysis, and contemporary HF biomarkers.^54^ By contrast, the larger study of acute coronary syndromes in a single coronary care unit of a Scandinavian teaching hospital (1268 patients; 70% men and 30% women) found CgA measured on day 1 to be an independent predictor of long‐term mortality and HF hospitalizations, as well as providing incremental prognostic information above evaluation of conventional cardiovascular risk factors.^50^ Of note, recurrent myocardial infarction was found not to be associated with plasma CgA levels after adjustments for conventional cardiovascular risk markers.^50^

## Full‐length CgA and vasostatins

A growing body of evidence suggests that full‐length CgA (hCgA_1–439_) and its N‐terminal fragments hCgA_1–76_ and hCgA_1–113_ are important players in the regulation of the cardiovascular system.^72^, ^73^ These fragments were named vasostatin‐1 and vasostatin‐2, respectively, because of their ability to suppress vasoconstriction in isolated human conduit vessels, originally observed.^22^, ^74^, ^75^, ^76^ After their discovery, a wide range of other biological effects were assigned to these polypeptides. For example, vasostatin‐1 has been implicated in the homeostatic regulation of plasma calcium, vascular tension, endothelial barrier integrity, innate immunity, inflammation, pain relief, intestinal motility, intestinal barrier, and cardiac functions.^55^, ^74^, ^76^, ^77^, ^78^ Full‐length CgA and vasostatin‐1 can modulate the adhesion of cardiomyocytes, smooth muscle cells, and fibroblasts, and to proteins involved in the extracellular matrix and endothelial cell–cell adhesion.^74^ Interestingly, in certain cases, full‐length CgA and fragments may exert opposite effects; for example, while vasostatin‐1 can enhance the adhesion of fibroblasts to extracellular matrix proteins, full‐length CgA inhibits cell adhesion.^25^

Studies performed with isolated and perfused rat hearts have shown that recombinant hCgA_1–439_ and vasostatin‐1 can depress myocardial contractility and relaxation, counteract the β‐adrenergic‐induced positive inotropism, and modulate coronary tone mainly via a nitric oxide–dependent mechanism.^72^, ^73^ Thus, these polypeptides can work as cardioregulatory hormones.

Vasostatin‐2, which includes the vasostatin‐1 (hCgA_1–76_) and the vasoconstrictive‐inhibitory factor sequence (hCgA_79–113_, known to act as a vasoregulatory molecule when liberated by cleavage^79^), increases coronary pressure in Langendorff‐perfused rat hearts without affecting inotropism, but can counteract the cardiostimulatory effects of isoproterenol.^44^ Using a rat model of myocardial infarction, other investigators have shown that vasostatin‐2 can improve cardiac function and reduce remodeling, fibrosis, and inflammation, suggesting that the reduced levels of this fragment observed in patients with ischemic CHF may have a pathogenic role.^58^

CgA and vasostatin‐1 can exert cardioprotective effects against ischemia/reperfusion (I/R) injury.^72^, ^73^ In this setting, vasostatin‐1 can act as a preconditioning inducer.^80^ Remarkably, hemodynamic and excitatory stimulation of rat hearts induce intracardiac CgA proteolytic processing to generate fragments containing the homologous vasostatin‐1 sequence.^81^ A recent study has shown that full‐length human CgA and vasostatin‐1 can also prevent cardiotoxic events induced by doxorubicin, a widely used anticancer drug whose clinical application is hampered by dose‐limiting cardiotoxicity.^82^ In particular, experimental evidence obtained in a rat model showed that low‐dose hCgA_1–439_ can protect the heart from inflammation, apoptosis, fibrosis, and ischemic injury caused by doxorubicin.^82^ Notably, this study also showed that pharmacologically active doses of hCgA_1–439_ do not impair the antitumor activity of doxorubicin in various murine models of solid tumors. Doxorubicin could also reduce both intracardiac expression and plasma levels of CgA.^82^ It appears, therefore, that doxorubicin can reduce the levels of this endogenous cardioprotective agent and that systemic administration of exogenous hCgA_1–439_ can restore cardioprotection. Interestingly, exogenous vasostatin‐1 could also exert cardioprotective effects on doxorubicin‐induced cardiotoxicity in the same rat model.^82^ However, in this case, a higher dose, compared with that necessary for hCgA_1–439_, was required to achieve cardioprotection, suggesting that the full‐length molecule is more potent. Monitoring CgA and vasostatin‐1 levels in cancer patients before and after doxorubicin therapy, the administration of low‐dose full‐length CgA to patients with low CgA levels might represent a novel approach to prevent doxorubicin‐induced cardiotoxicity, thereby meriting further investigation.

## Catestatin

Although initially identified as a physiological brake on catecholamine secretion,^27^, ^28^, ^29^, ^30^, ^31^, ^32^ CST is now established as a pleotropic hormone that reduces blood pressure,^33^, ^83^, ^84^, ^85^, ^86^, ^87^ positively regulates baroreflex sensitivity (BRS)^46^ and heart rate variability (HRV),^88^ and provides cardioprotection.^36^, ^39^, ^89^, ^90^, ^91^

### Vasodilatory and hypotensive effects of CST

**Figure 2:**
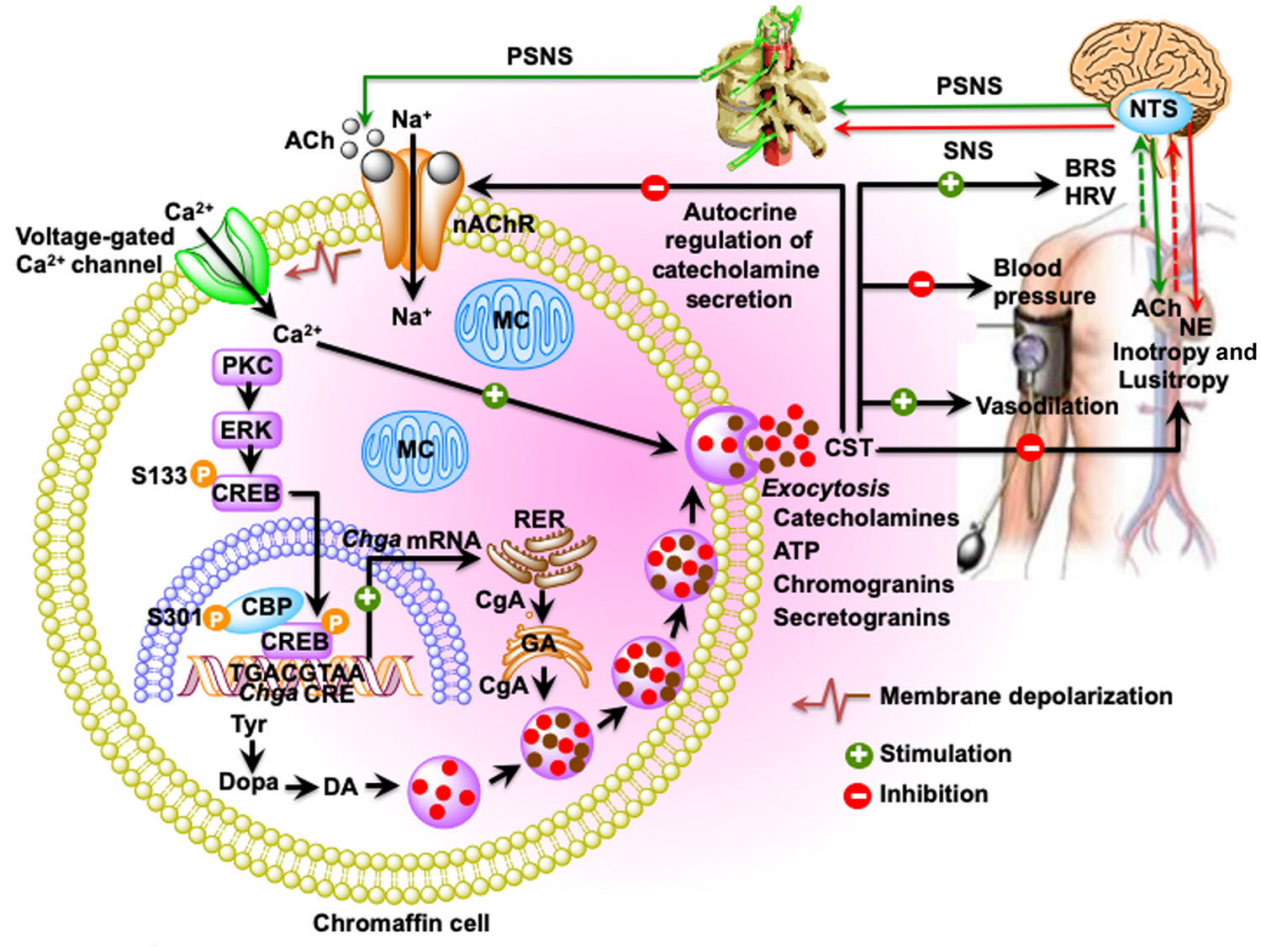
Schematic diagram showing the inhibition of catecholamine secretion and regulation of cardiovascular functions by CST. Acetylcholine released from preganglionic sympathetic endings at the splanchnic–adrenal synapse binds to nicotinic acetylcholine receptors and induces influx of Na^+^ inside chromaffin cells resulting in the depolarization of the cell membrane and opening of voltage‐gated Ca^2+^ channels. Increased cytosolic Ca^2+^ concentration stimulates the transcription of *Chga* and induces exocytotic release of the secretory cocktail containing catecholamines, CST, ATP, chromogranins, and neuropeptides. Exocytotically released CST inhibits further release of catecholamines by an autocrine/paracrine mechanism; decreases blood pressure and improves BRS and HRV by inhibiting catecholamine secretion; causes vasodilation by stimulating massive release of histamine; and decreases inotropy and lusitropy by activating the β_2_‐AR‐G_i/o_‐protein–PI‐3K–AKT–NO–cGMP signaling pathway. Ach, acetylcholine; BRS, baroreflex sensitivity; CA, catecholamine; CBP, CREB binding protein; CgA, chromogranin A protein; CREB, **cAMP‐responsive** element–binding protein; DA, dopamine; ERK, extracellular signal–regulated kinase; GA, Golgi apparatus; HRV, heart rate variability; MC, mitochondria; NE, norepinephrine; NTS, nucleus tractus solitarius; PKC, protein kinase C; PSNS, peripheral sympathetic nervous system; RER, rough endoplasmic reticulum; SNS, sympathetic nervous system; Tyr, tyrosine.

The hypotensive effects of CST (bovine CgA_344–364_) were first identified in pithed rats.^83^ Intravenous injection of bCST resulted in the reduction of pressor responses to the activation of sympathetic outflow by electrical stimulation. bCST also blunted neuropeptide Y agonist–induced hypertension. The vasodepressor effects of bCST appeared to be mediated by massive release of histamine (21‐fold increase) from mast cells, which is consistent with the vasodilatory effects of histamine.^92^ Using primary pleural and peritoneal cell populations containing 5% of mast cells, it was reported that bCST acts directly on mast cells to release histamine. The potency and efficacy of bCST were better evaluated when compared with the wasp venom peptide mastoparan.^93^ In human mast cell line LAD2, hCST induced migration of peripheral blood–derived mast cells to migrate, and caused degranulation and release of leukotriene C_4_ and prostaglandins D_2_ and E_2_;^94^ hCST also caused increased intracellular Ca^2+^ mobilization and induced the production of proinflammatory cytokines/chemokines, such as granulocyte‐macrophage colony–stimulating factor, chemokine C‐C motif ligand 2 (CCL2, also known as MCP1), chemokine C‐C motif ligand 3 (CCL3, also known as MIP1A), and chemokine C‐C motif ligand 4 (CCL4, also known as MIP1B).^94^ The vasodilatory effect of bCST was subsequently tested on human dorsal hand vein (to avoid systemic counterregulation) using hCST after pharmacologic vasoconstriction with phenylephrine, which showed dose‐dependent vasodilation, especially in female subjects;^86^ histamine‐induced vasodilation is believed to be mediated through increased production of nitric oxide. In rodents, the hypotensive effects of hCST were reported both in monogenic (hypertensive and hyperadrenergic *Chga*‐KO mice) and polygenic (blood pressure high mice) models of hypertension. In the monogenic model, hCST exerted its hypotensive effects by inhibiting the secretion of catecholamines^33^ (Fig. 2).

### Effects of CST on BRS and HRV

Consistent with diminished BRS in hypertensive subjects, hypertensive *Chga*‐KO mice also display diminished BRS.^46^ Intravenous administration of hCST increased BRS in hypertensive *Chga*‐KO mice, where both the reflex bradycardia caused by phenylephrine‐induced hypertension and the reflex tachycardia caused by sodium nitroprusside–induced hypotension were abolished or attenuated.^46^ These findings indicate that hCST resets the entire autonomic nervous reflex arc to restore normal cardiovascular function.^46^ Like BRS, hypertensive subjects display decreased HRV; and hypertensive *Chga*‐KO mice also exhibit decreased HRV.^88^ Intraperitoneal administration of hCST decreased total power (TP: ms^2^), which comprises low‐frequency power (LF: 0.4–1.5 Hz), high‐frequency power (HF: 1.5–4.0 Hz), and frequencies outside of low LF and HF power components, in the frequency domain of HRV spectra. hCST also improved each of the time domain parameters, such as mean difference in R–R between consecutive beats in ms (N–N), standard deviation of N–N in ms (SDNN), coefficient of variation of N–N (CVNN), % of consecutive beats more than 7 ms different in R–R (NN7%), reflecting cardiac parasympathetic tone, and root mean square of standard deviation of N–N in ms (RMSSD), which were decreased in *Chga*‐KO mice.^88^ Thus, hCST improves both BRS and HRV in *Chga*‐KO mice, which represents a monogenic model of rodent hypertension (Fig. 2).

### Central effects of CST

CgA is ubiquitously expressed in mouse,^30^, ^95^ rat,^96^, ^97^, ^98^ and sheep^99^ central nervous systems. More precisely, CgA is expressed in the cell bodies of most anatomically defined inhibitory, excitatory, and aminergic cell groups of the brain and spinal cord. Barosensitive, bulbospinal neurons in the rostral ventrolateral medulla (RVLM) provide the major tonic excitatory drive to the sympathetic neurons that maintain arterial pressure.^100^ Microinjection of a pharmacological dose of CST (rat CgA_367–387_; 50 nL, 1 mM dissolved in PBS) into the RVLM of vagotomized normotensive rat resulted in sympathoactivation of up to 22% with an increase in arterial blood pressure of up to 35 mmHg. rCST also increased sympathetic BRS with concomitant attenuation of somatosympathetic reflex, implicating rCST as a major player in the regulation of adaptive reflexes. The caudal ventrolateral medulla (CVLM), residing caudal to the RVLM, is the major source of the tonic GABAergic inhibition of presympathetic RVLM neurons. CVLM neurons inhibit immediate changes in blood pressure via the baroreflex and tonically blunt the activity of the presympathetic RVLM neurons by baroreceptor‐independent mechanisms. The later effects play an important role in determining the long‐term level of sympathetic vasomotor tone and blood pressure.^100^ Microinjection of rCST into the CVLM neurons of vagotomized normotensive rats caused excitation of CVLM neurons resulting in decreased mean arterial blood pressure (by ∼23%) and attenuation of sympathetic baroreflex (by ∼64%).^101^ By contrast, in spontaneously hypertensive (SHR) rats, infusion of hCST into the pyramidal neurons of the central amygdala exerted profound decrease in arterial blood pressure (by 65 mmHg).^102^ Like CVLM, central amygdaloid neurons are also GABAergic, send projections to RVLM, and inhibit excitatory outputs from RVLM with consequent decreases in arterial blood pressure. It thus appears that centrally CST acts as an excitatory peptide as opposed to its inhibitory action in the periphery.

### The role of CST in cardioprotection

The first indication that CST acts as a cardioprotective peptide came from a Langendorff‐perfused rat heart preparation study, where hCST inhibited both inotropy and lusitropy under basal and stimulated (β‐adrenergic and endothelin) conditions.^36^ These cardioprotective effects were shown to be mediated by the activation of the β_2_‐AR‐G_i/o_ protein–NO–cGMP pathways. In rat papillary muscles and isolated cardiomyocytes, hCST exerted antiadrenergic effects through the endothelial PI3K–AKT–eNOS pathway.^103^ It was later shown that hCST provides cardioprotection by stimulating phosphodiesterase type‐2 and NO‐dependent *S*‐nitrosylation.^39^ SHR rats mimic human CHF and display decreased Frank–Starling response.^104^ In both normotensive Wistar–Kyoto (WKY) and SHR rats, hCST caused a significant increase of the Frank–Starling response as evidenced by hCST‐induced increase in left ventricular pressure (LVP) and the maximal rate of LV contraction (LV *dP*/*dt* max) with greater effects in SHR rats. hCST also resulted in decreased myocardial relaxation of both WKY and SHR rats as evidenced by CST‐induced decrease in the maximal rate of LV relaxation (LV *dP*/*dt* min). A positive correlation was reported between hCST‐induced improvement in myocardial mechanical response and protein *S*‐nitrosylation. Thus, aged HF patients suffering from ventricular dilation or diminished autonomic heart modulation coupled with increased afterload and impaired Frank–Starling response could receive benefits from hCST.

**Figure 3:**
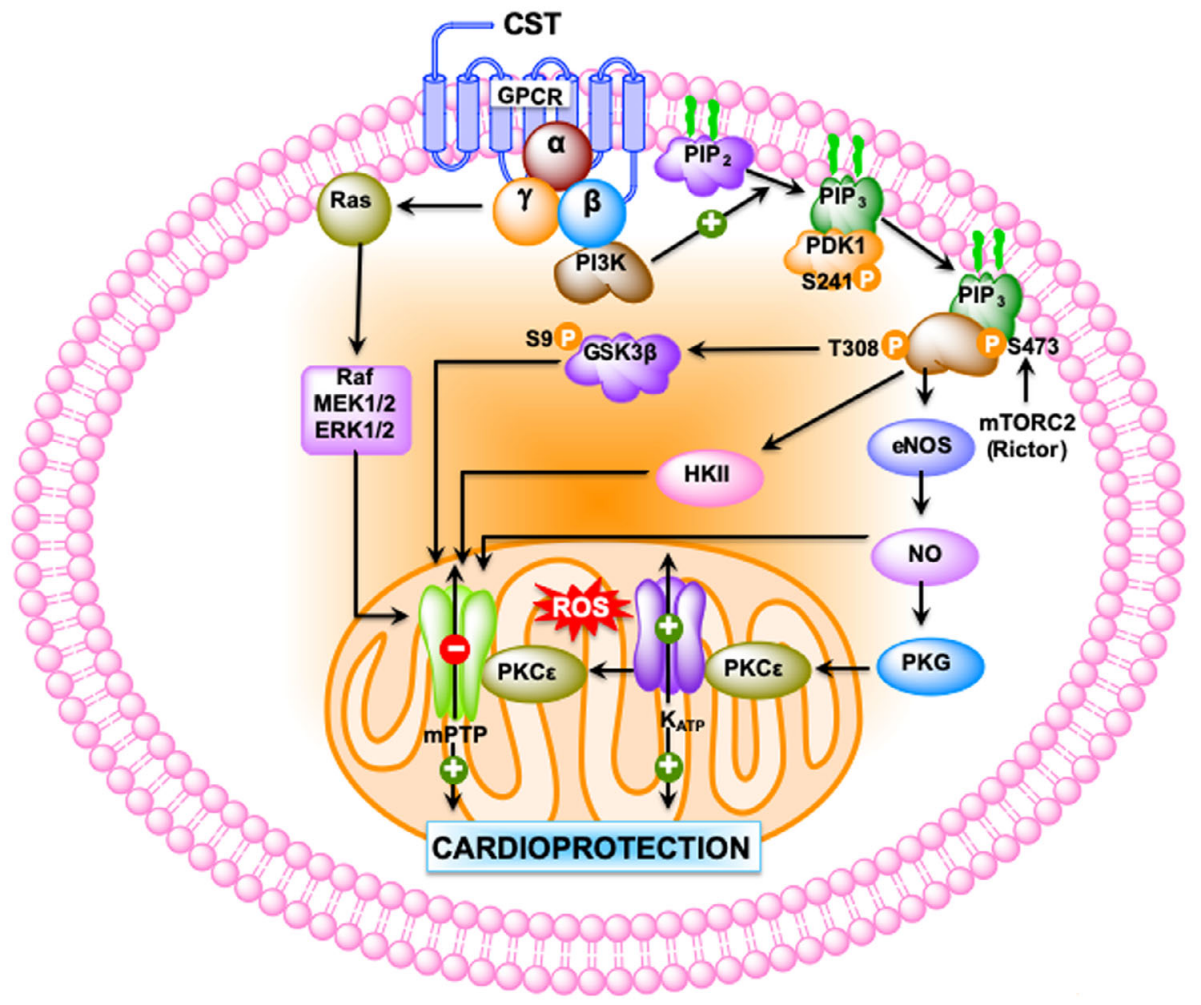
Schematic diagram showing the signaling pathways used by CST to provide cardioprotection. CST‐induced cardioprotection begins by the activation of G protein–coupled or cytokine receptors and the consequent recruitment of signaling pathways: (1) the reperfusion injury salvage kinase (RISK) pathway, including PI‐3K–AKT, ERK1/2, and the downstream target glycogen synthase kinase 3 beta (GSK‐3β); and (2) the PKG pathways. These salvage pathways activate downstream mediators, such as endothelial nitric oxide synthase (eNOS), GSK‐3β, hexokinase II (HKII), protein kinase C‐epsilon (PKCε), the mitochondrial ATP–dependent potassium channel (KATP) with consequent inhibition of mitochondrial permeability transition pore (MPTP). PDK1, pyruvate dehydrogenase kinase 1; ROS, reactive oxygen species.

hCST decreased I/R‐induced infarct size by ∼41%, which is comparable to the cardioprotection (∼44% decrease in infarct size) achieved through a postconditioning mechanism.^105^ Although data for attenuated I/R injury for hCST may seem promising, the data are based on experimental animal models that may not reflect human disease and previous clinical studies in the field have all had negative outcomes. Exogenous hCST (75 nM) at the onset of reperfusion resulted in attenuated postischemic rise of diastolic LVP (an index of contracture) and improved postischemic recovery of developed LVP. In addition, in isolated cardiomyocytes, hCST caused profound increase (by 65%) in cell viability after simulated I/R.^105^ Subsequently, it was shown that cardioprotection by hCST in early reperfusion stage was achieved by the activation of mitochondrial ATP‐sensitive K^+^  (mitoK_ATP_) channels, ROS signaling, and the prevention of mitochondrial permeability transition pore opening via upstream PI‐3K–AKT and PKC signaling^106^ (Fig. 3). In isolated rat cardiomyocytes undergoing simulated I/R, hCST increased cell survival 65% and decreased cell contracture of I/R cardiomyocytes. The maintenance of mitochondrial membrane potential in I/R cardiomyocytes and increased phosphorylation of AKT at S473, GSK3β at S9, PLB at T17, and eNOS at S1179 have been shown to play important roles in cardioprotection induced by hCST^107^ (Fig. 3).

### Effects of human variants of CST on rodent cardioprotection

Five single nucleotide polymorphisms have been discovered in the CST expressing region of *CHGA* in different human populations, namely, Gly364Ser (rs9658667), Pro370Leu (rs965868), Arg374Gln (rs9658669),^32^ Tyr363Tyr (rs9658666), and Gly367Val (rs200576557).^108^, ^109^ The cardiotropic actions of WT‐CST, Gly364Ser‐CST, and Pro370Leu‐CST were tested in isolated, Langendorff‐perfused rat heart preparation, demonstrating a dose‐dependent (11–200 nM) decrease in LVP (index of contractile activity), rate pressure product (index of cardiac work), and both positive and negative LV dP/dt (index of maximal rate of left ventricular contraction and relaxation, respectively), and increased coronary pressure by WT‐CST. Gly364Ser‐CST was ineffective on basal cardiac performance, and Pro370Leu‐CST induced only negative inotropic activity. Human CST variants also differed in the potencies with which they counteracted the positive inotropic and lusitropic effects of β‐adrenergic stimulation by ISO, with WT‐CST > Gly364Ser‐CST > Pro370Leu‐CST (against ISO‐induced positive inotropism) and Gly364Ser‐CST > WT‐CST > Pro370Leu‐CST (against ISO‐induced positive lusitropism).^36^

### Effects of Gly364Ser‐CST in BRS in humans

In humans, CST 364Ser variant caused profound changes in parasympathetic and sympathetic activity in individuals harboring the variant allele, which include an ∼47% and ∼44% increase in baroreceptor slope during upward and downward deflections, respectively, an ∼2.4‐fold increase in cardiac parasympathetic index, and an ∼26% decrease in cardiac sympathetic index in comparison with wild‐type individuals.^110^ It has been proposed that heightened baroreflex control of circulation may increase the longevity of 364S carriers. However, in response to cold stress, the blood pressure response was significantly less in the Gly/Ser heterozygotes when compared with the WT homozygotes.^110^

## Serpinin

In Langendorff‐perfused rat heart and papillary muscle preparations, both serpinin and pGlu‐serpinin cause dose‐dependent (11–165 nM) increase in cardiac contractility and relaxation, with pGlu‐serpinin being most potent and involving the β1 adrenoceptor–adenylate cyclase–cAMP–PKA pathway.^43^ This β1‐adrenergic agonist‐like activity of serpinin is in sharp contrast with the β2‐adrenoceptor depressive effects of vasostatin‐1 and CST. It thus appears that serpinin is either mimicking intracardiac sympathetic neurotransmitters and/or circulating catecholamines.^43^ Like CST, pGlu‐serpinin also provides cardioprotection in both pre‐ and postconditioning mechanisms in WKY and SHR rats. While in the preconditioning mechanism, pGlu‐serpinin caused ∼50% decrease in I/R‐induced infract size, it caused an ∼33% decrease in infarct size in a postconditioning mechanism of cardioprotection.^111^ How CST (β1‐adrenergic antagonist) and pGlu‐serpinin (β1‐adrenergic agonist) exerted comparable effects on cardioprotection by a postconditioning mechanism is yet to be addressed.

## Immunometabolic functions of catestatin

### Effects of CST on metabolism

The role of CST on metabolism came from a study in *Chga*‐KO mice, which are deficient in CST and PST and display obesity on a normal chow diet.^40^ Chronic hCST treatment was shown to decrease plasma triglyceride levels in *Chga*‐KO mice, which is believed to be due to increased lipolysis as evident from increased plasma glycerol and nonesterified fatty acids.^40^ This effect was mediated through inhibition of α_2_‐adrenergic receptor (α_2_‐AR) signaling. In *Chga*‐KO mice, hCST showed tissue‐specific effects on [^14^C]‐palmitate incorporation into lipids: decreased incorporation in adipose tissue, but increased incorporation in liver. By contrast, hCST‐induced oxidation of palmitate was comparable between adipose tissue and liver.^40^ Based on the above findings, it was concluded that hCST inhibits the expansion of adipose tissue and promotes fatty acid uptake in liver for β‐oxidation. Although hCST augmented expression of fatty acid oxidation genes, including carnitine palmitoyltransferase 1α (*Cpt1a*), peroxisome proliferator–activated receptor‐α (*Ppara*), acyl‐CoA oxidase 1 (*Acox1*), and uncoupling protein 2 (*Ucp2*), it exerted no effect on the expression of lipogenic genes, such as sterol regulatory element‐binding protein 1 (*Srebp1*) and peroxisome proliferator–activated receptor‐γ (*Pparg)*.^40^ In view of the above findings, it was concluded that hCST stimulates the incorporation of fatty acids into triglycerides, but not *de novo* lipogenesis.^40^ It thus appears that hCST mobilizes fat from adipose to liver for β‐oxidation. In diet‐induced obese and insulin‐resistant mice, hCST caused substantial decreases in hepatic triglyceride, NEFA, and ceramides concomitant with decreases in plasma TG and NEFA levels.^112^

### Effects on CST on immunometabolism

**Figure 4:**
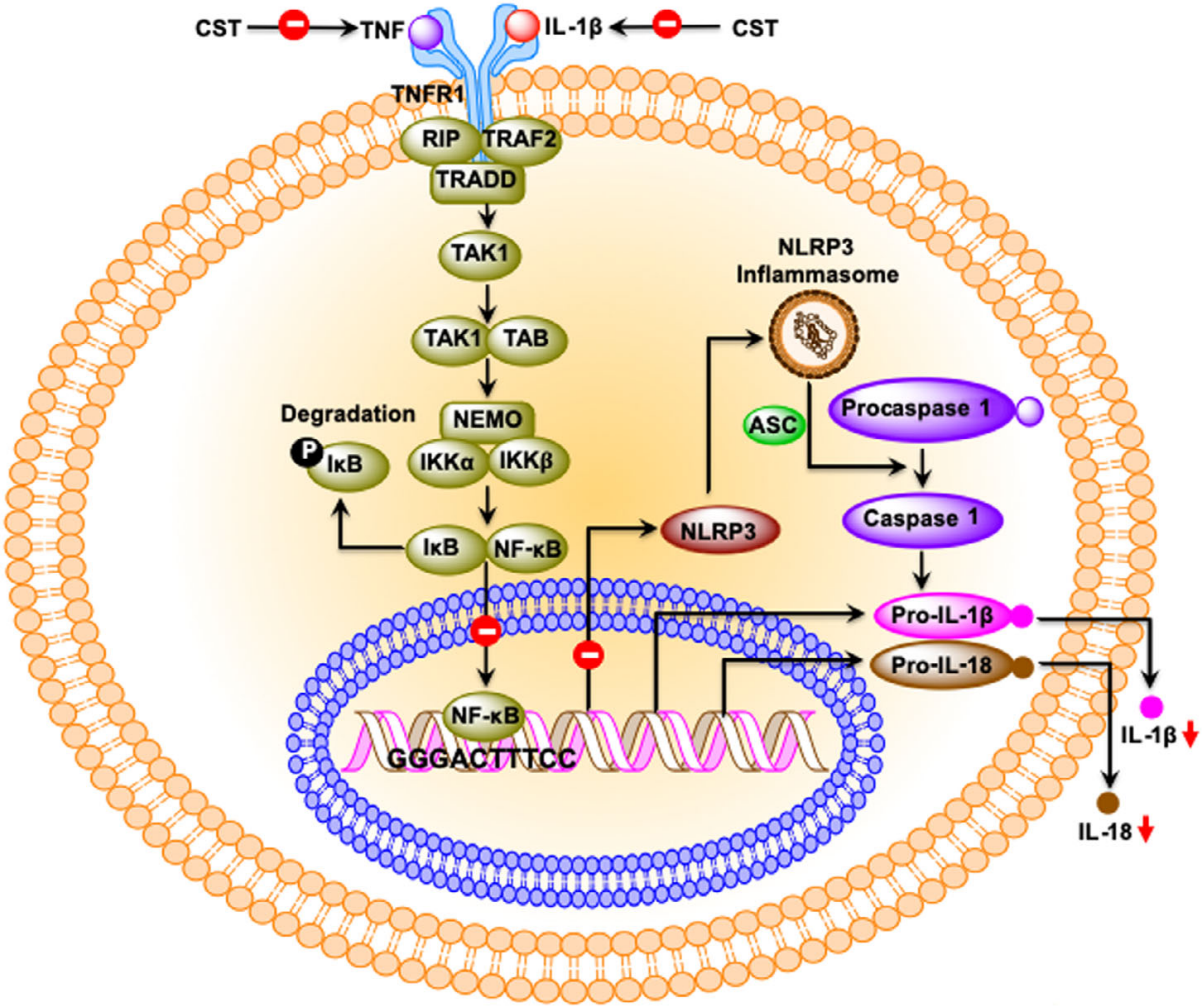
Schematic diagram showing the putative signaling pathways for the anti‐inflammatory actions of CST. Since CST decreases plasma TNF‐α levels, it is expected that less TNF‐α will be available for binding to its receptor (TNFR1), resulting in the attenuation of the IκB–NF‐κB signaling pathway leading to the translocation of fewer NF‐κB to the nucleus. This reduced NF‐κB signaling will affect the formation of NLRP3 inflammasome resulting in less cleavage procaspase 1 to active caspase 1 leading to less conversion of pro–IL‐1β and pro–IL‐18 to IL‐1β and IL‐18 and secretion of IL‐1β and IL‐18.

The term *immunometabolism* was coined in 2011 to describe the multifaceted interactions between the metabolic and immune systems.^113^ Both the innate (macrophages, eosinophils, neutrophils, and innate lymphoid type 1 and 2 cells)^114^, ^115^, ^116^, ^117^, ^118^, ^119^, ^120^, ^121^, ^122^, ^123^, ^124^ and adaptive (CD8^+^ T cells, CD3^+^CD4^+^ T helper type 1 and type 2 cells, CD3^+^CD4^+^FOXP3^+^ T regulatory cells, invariant natural killer T cells, and B lymphocytes)^125^, ^126^, ^127^, ^128^, ^129^, ^130^, ^131^, ^132^ immune cells control metabolism. The above studies were conducted in adipose tissues. The liver not only integrates nutrient, hormonal, and environmental signals to regulate glucose and lipid metabolism, but it also plays a key role as part of the immune system. It appears from the literature that cytokines, galectin‐3, and eicosanoids (LTB4) released from immune cells affect hepatocyte insulin signaling resulting in insulin resistance.^133^, ^134^, ^135^ TNF‐α, an inflammatory cytokine released from immune cells in the liver, potentiates steatosis by augmenting lipogenic activity and increasing biosynthesis of ceramides in liver.^136^ Increased intrahepatocyte ceramide levels in turn cause insulin resistance by inhibiting AKT signaling pathways.^137^ An intrahepatocyte proinflammatory program has also been shown to promote insulin resistance.^138^, ^139^ The first report on the role of CST on immunometabolism came from a recent study in diet‐induced obesity (DIO) and CST gene‐KO mice, where hCST was shown to improve insulin sensitivity by decreasing intrahepatic ceramide levels and inhibiting infiltration of monocyte‐derived macrophages (Ly6C^+^ proinflammatory macrophages) in liver. Chronic treatment of DIO mice with hCST resulted not only in the reduction of expression of proinflammatory genes (*Tnfa*, *Adgre1* (F4/80), *Itgam*, *Itgax*, *Ifng*, *Nos2*, and *Ccl2*), but also increased the expression of anti‐inflammatory genes (*Il10*, *MglI*, *Il4, Arg1*, and *Mrc1*) in both Kupffer cells (resident macrophages) and recruited monocyte–derived macrophages (LY6C^+^ macs) (Fig. 4). In addition, hCST caused significant reduction in proinflammatory cytokines, including IL‐1β and TNF‐α in plasma of DIO mice^112^ (Fig. 4). In human monocyte–derived THP‐1 cells, hCST also increased the expression of the anti‐inflammatory gene *Mrc1*.^140^

## Immunometabolic functions of PST

### Effects of PST on glucose‐stimulated insulin secretion

PST has been reported to inhibit glucose‐stimulated insulin secretion (GSIS) *in vivo* in mice, rats, dogs, and pigs, as well as *in vitro* from isolated rat islets.^141^ In the perfused rat pancreas model, PST (porcine CgA_240–288_) was shown to inhibit unstimulated and stimulated insulin secretion.^142^, ^143^, ^144^, ^145^ Consistent with the inhibitory role of PST in GSIS, GSIS was found to be ∼1.7‐fold higher in PST‐deficient *Chga*‐KO mice at 7 and 15 min after the administration of glucose.^45^ Although PST is reported to inhibit glucagon secretion induced by low glucose,^146^ it had no effect on somatostatin secretion.^147^ Besides the inhibition of GSIS, PST (hCgA_273–301_) also inhibits insulin‐stimulated glucose transport in primary rat and mouse adipocytes,^45^, ^148^, ^149^ differentiated 3T3‐L1 adipocytes,^150^, ^151^ and primary hepatocytes.^45^, ^152^

### Effects of PST on hepatic glucose metabolism

**Figure 5:**
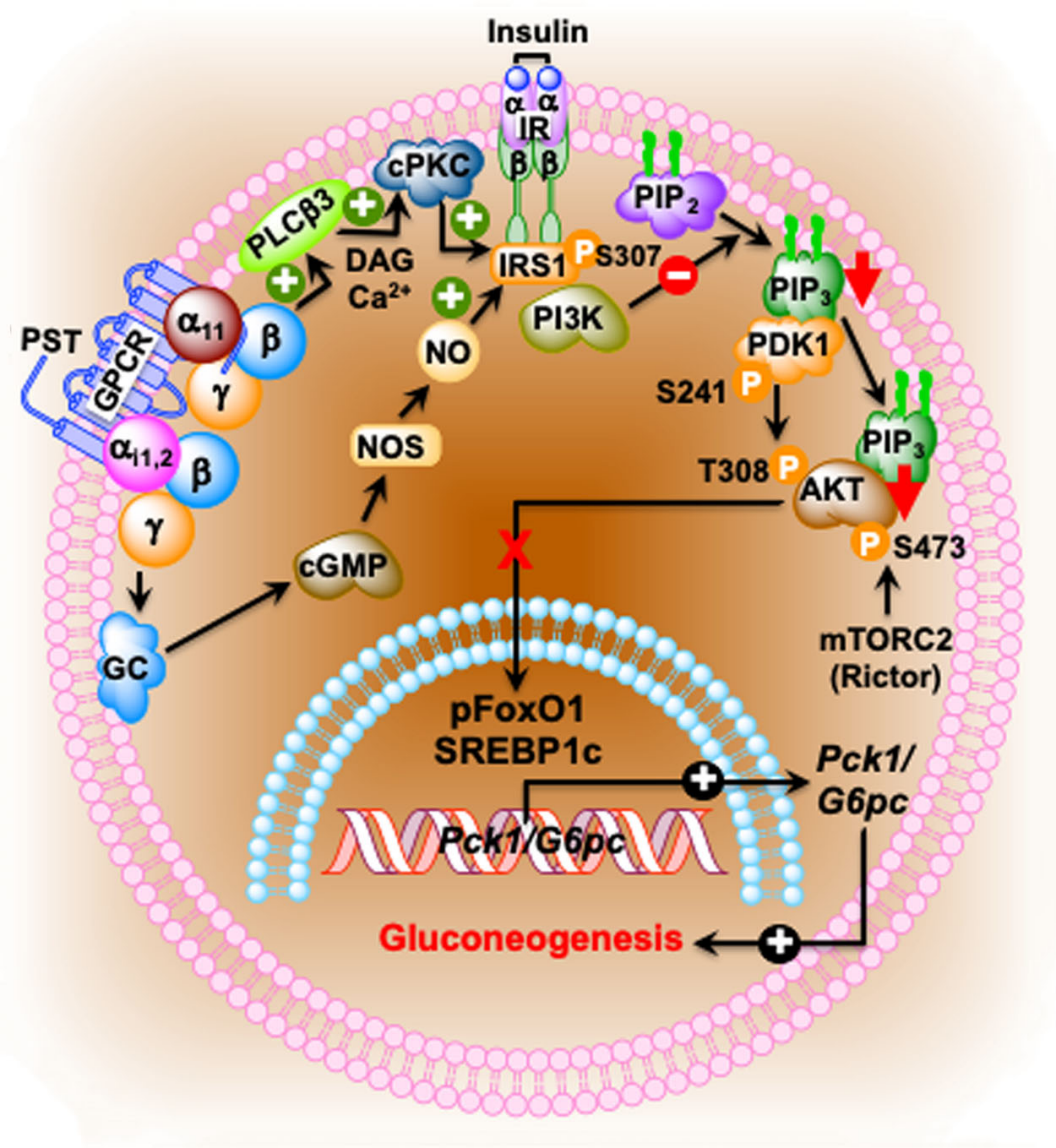
Schematic diagram showing the PST inhibition of gluconeogenesis in hepatocytes. PST initiates a GTP‐binding protein–linked signaling cascade, which results in the activation of diacylglycerol (DAG) and calcium‐dependent conventional PKC (cPKC), thereby attenuating IRS–PI3K–PDK1–AKT signaling pathway. In addition, the stimulation of the cGMP–NOS pathway also dampens this signaling pathway by nitrosylation of IRS. Suppression of this pathway by PST allows FoxO1 and SREBP1c to stimulate the expression of gluconeogenic genes, *Pck1* (also known as *Pepck*) and *G6pc* (also known as *G6Pase*), and thus prevents insulin action.

pPST has been shown to inhibit insulin‐stimulated glycogen synthesis in primary hepatocytes^153^ and activates glycogenolysis in the rat liver, which implicates a direct anti‐insulin effect on liver metabolism.^154^, ^155^ pPST decreases the phosphorylation of insulin receptor substrate 2 at tyrosine residues through the activation of conventional PKC, resulting in the activation of gluconeogenesis. Consistent with the anti‐insulin action, hPST also increases the production of NO with subsequent attenuated phosphorylation of protein kinase B (AKT), forkhead box protein O1 (FOXO1), and reduced matured sterol regulatory element‐binding transcription factor 1c (SREBP1c),^45^ thereby promoting gluconeogenesis (Fig. 5).

### Effects of PST on lipid metabolism

In addition to pronounced effects on glucose metabolism, PST also modulates lipid metabolism. PST (rat CgA_263–314_) decreases insulin‐stimulated synthesis of lipids in rat adipocytes.^156^ This finding is consistent with the hPST‐dependent increased expression of hepatic lipogenic genes in *Chga*‐KO mice, including *Srebp1c*, peroxisome proliferator‐activated receptor‐gamma (*Pparg*), and glycerol‐3‐phosphate acetyltransferase (*Gpat*).^45^ rPST has also been shown to stimulate the release of glycerol and free fatty acids from rat adipocytes, which is completely inhibited by insulin.^156^ In humans, hPST augments free fatty acid efflux into the circulation, resulting in an overall spillover of ∼4.5‐fold, which is consistent with the reported lipolytic action of rPST,^156^ confirming the anti‐insulin effects of PST.

### Effects of PST on inflammation and insulin resistance

The hallmarks of insulin resistance in DIO mice include obesity, hyperinsulinemia, and increased inflammation.^133^, ^157^, ^158^, ^159^, ^160^, ^161^ Therefore, the suppression of inflammation in DIO mice has been shown to improve insulin sensitivity.^162^, ^163^, ^164^ hPST treatment of *Chga*‐KO‐DIO mice resulted in increased expression of the proinflammatory genes, including interleukin‐1‐beta (*Il1b)*, TNF‐α (*Tnfa)*, interleukin‐6 *(Il6)*, chemokine C‐C motif ligand 2 *(Ccl2)*, and nitric oxide synthase 2a (*Nos2)*, thereby underscoring the proinflammatory nature of PST. Furthermore, the treatment of *Chga*‐KO–DIO mice with hPST caused significant reduction in the expression of *Arg1* and *Il10*. Consistent with gene expression data, *Chga*‐KO–DIO mice show decreased plasma levels of IL‐12p70, IFN‐γ, and chemokine C‐C motif ligand 3‐like 1 (CCL3L1), IL‐6, and chemokine C‐X‐C motif ligand 1 (CXCL1) compared with WT‐DIO mice.

## Regulation of angiogenesis and tumor growth

### Circulating CgA in patients with neuroendocrine and nonneuroendocrine tumors

Experimental evidence suggests that physiological levels of circulating CgA and its fragments contribute to regulate the vascular physiology in normal and neoplastic tissues.^165^ CgA is exocytotically released in circulation by the neuroendocrine system to reach, in healthy subjects, about 0.2–1 nM levels.^165^ This fact implies that tumors with mature and functional blood vessels, either neuroendocrine or nonneuroendocrine, are exposed to physiological amounts of blood‐borne CgA mainly derived from the diffuse neuroendocrine system. Furthermore, although many patients show normal levels of plasma CgA, some patients may have higher levels for a variety of reasons. For example, patients treated with proton pump inhibitors—a class of drugs known to induce gastric enterochromaffin‐like cell hyperplasia and CgA production—may have increased levels of circulating CgA.^166^, ^167^, ^168^, ^169^ Increased levels of immunoreactive CgA have been also detected in the blood of patients with renal failure, HF, hypertension, atrophic gastritis, inflammatory bowel disease, rheumatoid arthritis, sepsis, and other inflammatory diseases.^55^ Thus, in patients with nonneuroendocrine neoplasms, the tumor vasculature is exposed to variable levels of CgA derived from the diffuse neuroendocrine system.

The situation is even more complex in the case of neuroendocrine tumors or tumors with neuroendocrine differentiation, as in both these cases the tumor vasculature can be exposed not only to CgA molecules released by neuroendocrine system into the blood, but also to CgA released in tumor microenvironments (and then into the blood) by neoplastic cells. Indeed, cancer cells in neuroendocrine tumors (including pheochromocytomas, carcinoid tumors of the intestine, lung and stomach, parathyroid and medullary thyroid carcinomas, neural tumors, anterior pituitary tumors, small cell lung cancer, pancreaticoduodenal tumors, and many others) can express and secrete CgA in relatively large amounts.^167^ Also, tumors presenting the focal expression of CgA consequent to neuroendocrine differentiation (such as breast and prostate cancer, non‐small cell lung cancer, and gastric and colonic adenocarcinomas) may also release CgA.^7^, ^167^ Thus, the tumor vasculature, in these cases, might be affected by CgA present in the blood and by tumor‐derived CgA in very complex ways.

In this regard, it is important to highlight the fact that detection of full‐length CgA and fragments using specific ELISA systems based on selective antibodies has shown that CgA, either systemically or locally released, consists of variable mixtures of intact protein and fragments, including full‐length CgA (hCgA_1–439_), the vasostatin‐1 fragment hCgA_1–76_, fragments lacking part or the entire C‐terminal region, such as hCgA_1–373_, and other shorter peptides.^41^, ^170^ For example, hCgA_1–373_, which is either not or minimally present in the blood of healthy subjects, is increased in peripheral blood and bone marrow plasma of patients with multiple myeloma, while full‐length CgA is decreased in these patients.^170^ A central issue is whether changes in the circulating levels of full‐length CgA and its fragments (absolute or relative), which may occur in cancer patients, are epiphenomena of the presence of the tumors or are contribute to tumor pathophysiology. In the following sections, we will discuss the experimental evidence suggesting that CgA and its fragments contribute to regulate tumor growth and responses to therapy.

### Role of full‐length CgA and vasostatin‐1 in tumor angiogenesis and growth

It is well known that the unbalanced production of anti‐ and proangiogenic factors in tumors can lead to vascular abnormalities that contribute to tumor cell proliferation, invasion, trafficking, and metastasis formation.^171^, ^172^, ^173^ CgA may be one of the factors that contributes to regulate tumor vascular biology.^76^, ^165^ For example, recent studies have shown that full‐length CgA (hCgA_1–439_) can function as an antiangiogenic factor, with a functional antiangiogenic site in the C‐terminal region 410–439 and a latent (or less active) site in the N‐terminal region 1–76 (vasostatin‐1), which requires proteolytic cleavage for full activation.^41^ Full‐length CgA and vasostatin‐1 can inhibit endothelial cell migration, motility, invasion, and capillary‐like structure formation induced by vascular endothelial growth factor (VEGF) or fibroblast growth factor‐2 (FGF2), two important proangiogenic factors.^41^, ^174^ Vasostatin‐1 can also inhibit the nuclear translocation of hypoxia inducible factor‐1α, a master regulator of angiogenesis, in endothelial cells.^175^ These proteins can enhance the endothelial barrier function and reduce vascular leakage and trafficking of tumor cells through the endothelium.^176^, ^177^

Studies performed in mice have shown that endogenous murine CgA or exogenous recombinant hCgA_1–439_, but not the fragments lacking the C‐terminal region, can inhibit tumor growth in models of nonneuroendocrine solid‐tumors, including fibrosarcoma, mammary adenocarcinoma, Lewis lung carcinoma, and primary and metastatic melanoma.^178^ The inhibition of tumor growth is associated with reduction of microvessel density and blood flow in neoplastic tissues. Also, low‐dose vasostatin‐1 can delay tumor growth and reduce microvessel density in murine models of solid tumors.^41^ In these models, neutralization of endogenous murine CgA with antibodies against its C‐terminal region enhances tumor growth rate, whereas systemic administration of low amounts of hCgA_1–439_ reduces tumor growth.^178^ It appears, therefore, that circulating full‐length CgA and vasostatin‐1 can work as physiological inhibitors of tumor growth.

**Figure 6:**
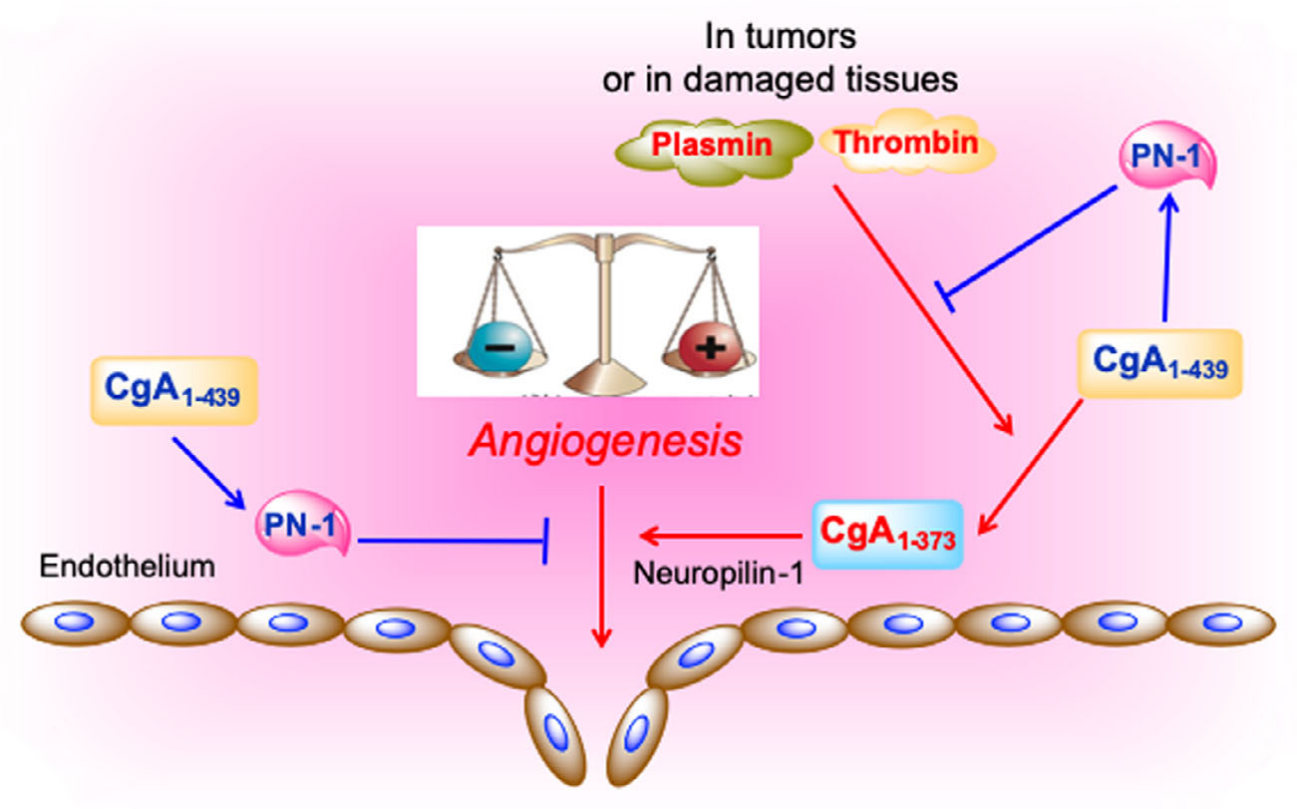
Hypothetical model of the CgA‐dependent angiogenic switch. According to this model, in normal conditions, full‐length CgA_1–439_ exerts antiangiogenic effects by inducing protease nexin‐1 (PN‐1), an antiangiogenic protein. Since PN‐1 is also a potent inhibitor of plasmin and thrombin, this protein can also prevent CgA cleavage by these enzymes and preserve its antiangiogenic activity. In damaged tissues or tumors, the balance of protease/antiprotease activity is altered leading to CgA cleavage and formation of fragments, for example, CgA_1–373_, capable of interacting with neuropilin‐1 on endothelial cells (via its PGPQLR sequence) and stimulating angiogenesis (see text).

Mechanistically, full‐length CgA induces the production of protease nexin‐1 in endothelial cells, a serine protease inhibitor (serpin) that can inhibit endothelial cell migration, capillary tube formation, and angiogenesis, independently of its serpin activity^179^, ^180^ Given that the neutralization of protease nexin‐1 with specific antibodies can suppress the antiangiogenic and antitumor effects of recombinant hCgA_1–439_, it is possible that the induction of protease nexin‐1 by CgA, as observed in animal models, is an important mechanism underlying its antiangiogenic and antitumor activities (Fig. 6). Furthermore, considering that protease nexin‐1 is also a potent inhibitor of plasmin and thrombin,^181^ two proteolytic enzymes often present in tumors, and that CgA can be cleaved by these enzymes,^41^, ^170^ this inhibitor might also serve to prevent the local cleavage of CgA, thereby preserving its antiangiogenic activity (Fig. 6). In view of the inhibitory activity of circulating full‐length CgA on angiogenesis and tumor growth, and considering that cleavage of its C‐terminal region markedly reduces its activity, it is reasonable to hypothesize that pathophysiological changes of circulating levels of full‐length CgA and/or its fragmentation may contribute to the regulation of disease progression in patients with non‐neuroendocrine tumors.

CgA typically affects tumor growth with a U‐shaped dose–response curve.^178^ This implies that a marked increase of its concentration may paradoxically result in a reduced effect. For this reason, the effects of CgA on neuroendocrine tumors could be different from those observed in murine models of nonneuroendocrine tumors (and difficult to predict), considering the high concentration that CgA can reach in the tumor microenvironment because of the secretory activity of neuroendocrine cancer cells. Furthermore, one cannot exclude the possibility that the tumor‐derived CgA might be fragmented in a different manner, compared with CgA released in the blood by the neuroendocrine system. Thus, although CgA is a widely used serological and histopathological marker for neuroendocrine tumor diagnosis and follow‐up, development of appropriate models is necessary to address the functional role of CgA and its fragments in patients with such tumors.

### Role of CgA_1–373_ in tumor angiogenesis and growth

Recent studies have shown that a human CgA fragment encompassing residues 1–373 (hCgA_1–373_; thus lacking the C‐terminal region) promotes angiogenesis by inducing the release of FGF2 from endothelial cells.^35^, ^41^, ^182^ Notably, hCgA_1–373_, hCgA_1–76_, and hCgA_1–439_ can counterbalance the activity of each other in angiogenesis assays,^183^ suggesting that these proteins may provide a balance of pro‐ and anti‐angiogenic activities tightly regulated by proteolysis. The transition from anti‐ to pro‐angiogenic forms, which can be induced by limited digestion of CgA with plasmin or thrombin,^41^, ^183^ occurs in the bone marrow of multiple myeloma patients and correlates with increased tumor microvessel density.^170^

Studies aimed at elucidating the proangiogenic mechanisms of hCgA_1–373_ and its role in tumor progression have shown that cleavage of the R_373_R_374_ dibasic site of CgA leads to the exposure of the PGPQLR_373_ epitope (100% conserved in mouse and human CgA), and that this is crucial for tumor progression in various murine models of nonneuroendocrine tumors.^184^ Indeed, blockade of the PGPQLR site of murine CgA with specific antibodies, unable to recognize full‐length CgA, inhibits tumor growth in various experimental models.^184^ Blockade of the PGPQLR site with antibodies induces changes in the tumor vascular bed and blood flow in murine fibrosarcomas, suggesting that circulating CgA might work as an “off/on” switch for tumor angiogenesis, when cleaved at the PGPQLR site. Thus, fragmentation of circulating CgA in tumors by local proteases may contribute to “switch on” angiogenesis, and changes in the balance of protease/antiprotease molecules in tumor tissues might represent major mechanisms for regulating the CgA activity (Fig. 6).

Mechanistic studies have shown that hCgA_1–373_, but not hCgA_1–439_, can bind neuropilin‐1 with good affinity (*K*_d_ = 3.49 ± 0.73 nM), suggesting that this protein is an important receptor for hCgA_1–373_. In particular, the PGPQLR site of hCgA_1–373_, but not that of hCgA_1–439_, can bind the VEGF binding‐site of neuropilin‐1.^184^ It appears, therefore, that the PGPQLR binding site is cryptic in the full‐length molecule or that this region undergoes profound conformational changes upon cleavage of CgA at the R_373_R_374_ dibasic site. No binding to neuropilin‐1 occurs with hCgA_1–372_, a fragment lacking R_373_, suggesting that the C‐terminal arginine of PGPQLR site (R_373_) is crucial for binding^184^ (Fig. 6). Considering that the interaction between fragmented CgA and neuropilin‐1 is important for tumor growth, the PGPQLR site may be a potential therapeutic target.

Neuropilin‐1 is an important coreceptor of various protein factors that regulate vascular permeability and angiogenesis, such as semaphorins and VEGF. The C‐terminal sequence of VEGF (CDKPRR), which contains an R/K‐X‐X‐R/K C‐end rule motif (CendR), can recognize a pocket in the b1 domain of neuropilin‐1, a site that can also accommodate other peptides containing the CendR motif;^185^, ^186^ in addition, the PGPQLR site of hCgA_1–373_ can interact with this pocket of neuropilin‐1.^184^ Of note, while both hCgA_1–373_ and VEGF can bind neuropilin‐1 with similar affinity, only VEGF can bind neuropilin‐2.^184^

It is possible that hCgA_1–373_ engages other coreceptors for signaling. The hCgA_352–372_ sequence (catestatin), for example, is known to bind the acetylcholine nicotinic receptor (nAChRs).^85^, ^187^, ^188^ Considering that this class of receptors is expressed on endothelial cells and known to regulate angiogenesis,^189^, ^190^, ^191^, ^192^ nAChRs might be potential targets. This view is supported by the observation that mecamylamine and α‐bungarotoxin, two nAChRs antagonists, can inhibit the proangiogenic activity of hCgA_1–373_ in a human umbilical vein endothelial cell (HUVEC) spheroid assay.^184^ Thus, these receptors, in addition to neuropilin‐1, might play a role in the proangiogenic activity of hCgA_1–373_.

The R_373_ residue of hCgA_1–373_ can be rapidly removed by a plasma carboxypeptidase,^184^ resulting in the loss of neuropilin‐1 binding and proangiogenic activity and gain of antiangiogenic activity. Interestingly, subnanomolar concentrations of hCgA_1–372_ (antiangiogenic) can inhibit the activity of subnanomolar levels of hCgA_1–373_ (proangiogenic), but not that of VEGF and FGF2.^184^ Thus, the transition of hCgA_1–373_ to hCgA_1–372_ may be a specific counter‐regulatory mechanism that serves to limit the activity of hCgA_1–373_ at the site of production in tumors. Another potential counter‐regulatory mechanism induced by an excess of hCgA_1–373_ could be related to its biphasic behavior, as suggested by its bell‐shaped dose–response curve observed in angiogenesis assays.^41^, ^178^, ^184^ The mechanisms underlying this behavior are unknown.

In summary, the cleavage of the R_373_R_374_ site of CgA in tumors and the subsequent removal of R_373_ in plasma may provide an important on/off switch for spatiotemporal regulation of angiogenesis in tumors and normal tissues. The interaction of the PGPQLR site with neuropilin‐1 might be, therefore, an important target for angiogenesis inhibition and tumor growth suppression.

### Role of full‐length CgA and vasostatin‐1 in tumor cell trafficking

Cancer progression involves the seeding of malignant cells in circulation and the colonization of distant organs. Circulating neoplastic cells can also reinfiltrate the tumor of origin in a process referred to as *tumor self‐seeding*.^177^ A study performed in murine models of solid tumors has shown that a modest increase of circulating CgA, as obtained in mice after the administration of omeprazole (a proton pump inhibitor) or low‐dose recombinant hCgA_1–439_, can reduce tumor self‐seeding and dissemination.^177^ In particular, the study showed that hCgA_1–439_ can inhibit shedding of cancer cells in circulation, tumor reinfiltration by circulating cells, and lung colonization in murine models of mouse mammary adenocarcinomas and melanomas. Mechanistic studies showed that hCgA_1–439_ can reduce endothelial cell gap formation and vascular leakage induced by tumor‐derived factors in tumors, as well as the transendothelial migration of cancer cells.^176^, ^177^ Thus, circulating full‐length CgA, besides reducing angiogenesis in tumors, can enhance endothelial barrier function in both tumors and normal tissues thereby inhibiting the trafficking of tumor cells via tumor–to–blood and blood–to–tumor/normal tissue routes (i.e., metastasis and tumor self‐seeding processes).^176^, ^177^ Similar effects have been reported for vasostatin‐1.^177^

A role for circulating CgA in cancer cell trafficking has also been investigated in chronic lymphocytic leukemia (CLL), a hematological cancer characterized by the progressive accumulation of CD5^+^ leukemic B cells in peripheral blood, lymphoid tissues, and bone marrow, and by the continuous trafficking of these cells among these tissue compartments.^193^ Physiologically relevant doses of recombinant hCgA_1–439_ have been shown to reduce the bone marrow/blood ratio of leukemic cells in the Eµ‐TCL1 murine transgenic CCL model, and decrease bone marrow, kidney, and lung infiltration in *Rag2* ^−/−^ γc (*Il2rg*)^−/−^ mice engrafted with human MEC1 CLL cells.^170^ This treatment also reduced the loss of body weight associated with disease progression and improved animal motility. hCgA_1–439_ was found to enhance the integrity of the endothelial barrier and to reduce the transendothelial migration of MEC1 cells with a bimodal dose–response curve. Vasostatin‐1, but not hCgA_1–373_, could inhibit CLL progression in the xenograft model, suggesting that the N‐terminal domain contains a site activated by proteolytic cleavage, and that the C‐terminal region is crucial for CgA activity.^183^ It is therefore possible that CgA and its fragments contribute to regulate leukemic cell trafficking and tissue infiltration in patients with CLL.

### Full‐length CgA and response to anticancer therapy

The efficacy of chemotherapy in cancer patients is often limited by inadequate and uneven penetration of drugs in tumor tissues.^194^, ^195^ This phenomenon depends on the abnormal vasculature and altered tissue composition typically present in solid tumors, which may cause irregular blood flow, heterogeneous permeability, increased interstitial fluid pressure, vascular compression and, consequently, uneven drug penetration.^195^, ^196^, ^197^ We have shown that NGR‐TNF, a derivative of TNF‐α that targets tumor blood vessels and enhances vascular permeability, improves drug diffusion into neoplastic tissues in various animal models and patients.^197^, ^198^, ^199^, ^200^ A recent study in patients with primary lymphomas of the central nervous system has shown that this drug can break the blood–brain barrier in tumor and peritumoral areas and enhance the response to chemoimmunotherapy.^201^

Studies performed in murine lymphoma and melanoma models have shown that pathophysiologically relevant doses of hCgA_1–439_ are sufficient to reduce the NGR‐TNF–induced penetration of doxorubicin in tumors by enhancing the endothelial barrier function.^177^ *In vitro* studies, performed with human endothelial cell monolayers, showed that hCgA_1–439_ can inhibit the disassembly of VE‐cadherin dependent adherence junctions and permeability to drugs induced by NGR‐TNF. Similar effects in animal models were observed with vasostatin‐1.^177^ *In vitro*, this fragment can inhibit various effects exerted by TNF‐α on endothelial cells, such as p38‐MAP kinase phosphorylation, VE‐cadherin redistribution, gap formation, VEGF‐ and thrombin‐induced endothelial cell permeability, and paracellular transport of macromolecules.^174^, ^176^, ^202^, ^203^ Thus, both full‐length CgA and vasostatin‐1 are efficient inhibitors of TNF‐α–induced alteration of the endothelial barrier function and permeability.

It is therefore possible that increased levels of circulating CgA and its fragments, as may occur in the subpopulation of cancer patients with non‐neuroendocrine tumors, can reduce drug delivery to tumor cells, particularly when they are combined with NGR‐TNF. Of note, daily treatment of mice with omeprazole, a proton pump inhibitor commonly used in the treatment of acid peptic disorders, increased the circulating levels of CgA by twofold and inhibited the penetration of doxorubicin induced by NGR‐TNF in tumors.^177^ Thus, even a modest enhancement of CgA levels is sufficient to inhibit the synergism of NGR‐TNF with chemotherapy. Considering the wide use of proton pump inhibitors in patients, these findings may have important clinical implications. Therefore, monitoring CgA and its fragments might help to select patients who respond better to this combination therapy. Discontinuation of administration of proton pump inhibitors to patients and/or its replacement with H_2_ receptor antagonists might increase the response rate in patients with elevated levels of circulating CgA or vasostatin‐1.

## Conclusions

The CgA system, comprising full‐length CgA and its fragments, is emerging as an important player in cardiovascular, immunometabolic, and cancer regulation. A major gap of knowledge in this field is related to the receptor mechanisms of the diverse biological functions of the CgA‐related polypeptides, with few exceptions regarding the direct interaction of human CgA with the integrin α_v_/β_6_ in the regulation of keratinocyte adhesion,^204^ CST with nicotinic acetylcholine receptors in the inhibition of catecholamine secretion by chromaffin cells,^27^, ^31^, ^187^, ^188^, ^205^, ^206^ and hCgA_1–373_ with neuropilin‐1 in angiogenesis and tumor growth stimulation.

In this regard, it is important to highlight the fact that most of the information on the biological functions of CgA and its fragments has been obtained with experiments performed with human‐derived molecules in mouse or rat *in vivo* models, with only few exceptions. Although the regions corresponding to the main bioactive fragments of CgA show high degree of sequence similarity among different species, the sequences are not identical, introducing the possibility that different receptor mechanisms are brought into play. For example, while human CgA contains an RGD site that can interact with high selectivity and affinity with the integrin α_v_/β_6_, this site is changed with QGD in mouse and rat CgA, with loss of integrin binding.^204^ Furthermore, most experiments with full‐length CgA have been performed with hCgA_1–439_ produced by recombinant DNA technology in bacterial cells, and thus lacking potentially important posttranslational modifications. These points are potential important limitations of studies performed thus far and may explain some of the controversial results obtained in some cases. Further experiments with syngeneic polypeptides and animal models are necessary to fully elucidate the activity of the CgA system in normal and pathological conditions.

Nevertheless, data have highlighted the complexity of the CgA system in cardiovascular, immunometabolic, and cancer regulation and suggest that qualitative and quantitative characterization of CgA and all its fragments, in normal subjects and diseased patients, are necessary to fully assess the biological role of this system and its potential diagnostic/prognostic value.

## Competing interests

The authors declare no competing interests.

## References

[1] Banks, P. & K. Helle . 1965 The release of protein from the stimulated adrenal medulla. Biochem. J. 97: 40C–41C. 10.1042/bj0970040c PMC1264782 5881651

[2] Blaschko, H. , R.S. Comline , F.H. Schneider , *et al* 1967 Secretion of a chromaffin granule protein, chromogranin, from the adrenal gland after splanchnic stimulation. Nature 215: 58–59. 6053402 10.1038/215058a0

[3] Schneider, F.H. , A.D. Smith & H. Winkler . 1967 Secretion from the adrenal medulla: biochemical evidence for exocytosis. Br. J. Pharmacol. Chemother. 31: 94–104. 6058830 10.1111/j.1476-5381.1967.tb01980.x PMC1557278

[4] Konecki, D.S. , U.M. Benedum , H.H. Gerdes , *et al* 1987 The primary structure of human chromogranin A and pancreastatin. J. Biol. Chem. 262: 17026–17030. 2445752

[5] Winkler, H. & R. Fischer‐Colbrie . 1992 The chromogranins A and B: the first 25 years and future perspectives. Neuroscience 49: 497–528. 1501763 10.1016/0306-4522(92)90222-N PMC7131462

[6] Iacangelo, A.L. & L.E. Eiden . 1995 Chromogranin A: current status as a precursor for bioactive peptides and a granulogenic/sorting factor in the regulated secretory pathway. Regul. Pept. 58: 65–88. 8577930 10.1016/0167-0115(95)00069-n

[7] Taupenot, L. , K.L. Harper & D.T. O'Connor . 2003 Mechanisms of disease: the chromogranin‐secretogranin family. N. Engl. J. Med. 348: 1134–1149. 12646671 10.1056/NEJMra021405

[8] Mosley, C.A. , L. Taupenot , N. Biswas , *et al* 2007 Biogenesis of the secretory granule: chromogranin A coiled‐coil structure results in unusual physical properties and suggests a mechanism for granule core condensation. Biochemistry 46: 10999–11012. 17718510 10.1021/bi700704r

[9] Metz‐Boutigue, M.H. , P. Garcia‐Sablone , R. Hogue‐Angeletti , *et al* 1993 Intracellular and extracellular processing of chromogranin A. Determination of cleavage sites. Eur. J. Biochem. 217: 247–257. 8223562 10.1111/j.1432-1033.1993.tb18240.x

[10] Taylor, C.V. , L. Taupenot , S.K. Mahata , *et al* 2000 Formation of the catecholamine release‐inhibitory peptide catestatin from chromogranin A. Determination of proteolytic cleavage sites in hormone storage granules. J. Biol. Chem. 275: 22905–22915. 10781584 10.1074/jbc.M001232200

[11] Lee, J.C. , C.V. Taylor , S.P. Gaucher , *et al* 2003 Primary sequence characterization of catestatin intermediates and peptides defines proteolytic cleavage sites utilized for converting chromogranin a into active catestatin secreted from neuroendocrine chromaffin cells. Biochemistry 42: 6938–6946. 12795588 10.1021/bi0300433

[12] Biswas, N. , J.L. Rodriguez‐Flores , M. Courel , *et al* 2009 Cathepsin L co‐localizes with chromogranin a in chromaffin vesicles to generate active peptides. Endocrinology 150: 3547–3557. 19372204 10.1210/en.2008-1613 PMC2717865

[13] Parmer, R.J. , M. Mahata , Y. Gong , *et al* 2000 Processing of chromogranin A by plasmin provides a novel mechanism for regulating catecholamine secretion. J. Clin. Invest. 106: 907–915. 11018079 10.1172/JCI7394 PMC381423

[14] Jiang, Q. , L. Taupenot , S.K. Mahata , *et al* 2001 Proteolytic cleavage of chromogranin A (CgA) by plasmin: selective liberation of a specific bioactive CgA fragment that regulates catecholamine release. J. Biol. Chem. 276: 25022–25029. 11342539 10.1074/jbc.M101545200

[15] Biswas, N. , S.M. Vaingankar , M. Mahata , *et al* 2008 Proteolytic cleavage of human chromogranin a containing naturally occurring catestatin variants: differential processing at catestatin region by plasmin. Endocrinology 149: 749–757. 17991725 10.1210/en.2007-0838 PMC2219303

[16] Benyamin, B. , A.X. Maihofer , A.J. Schork , *et al* 2017 Identification of novel loci affecting circulating chromogranins and related peptides. Hum. Mol. Genet. 26: 233–242. 28011710 10.1093/hmg/ddw380 PMC6075630

[17] Tatemoto, K. , S. Efendic , V. Mutt , *et al* 1986 Pancreastatin, a novel pancreatic peptide that inhibits insulin secretion. Nature 324: 476–478. 3537810 10.1038/324476a0

[18] Sanchez‐Margalet, V. , C. Gonzalez‐Yanes , S. Najib , *et al* 2010 Metabolic effects and mechanism of action of the chromogranin A‐derived peptide pancreastatin. Regul. Pept. 161: 8–14. 20184923 10.1016/j.regpep.2010.02.005

[19] Curry, W.J. , C. Shaw , C.F. Johnston , *et al* 1992 Isolation and primary structure of a novel chromogranin A‐derived peptide, WE‐14, from a human midgut carcinoid tumour. FEBS Lett. 301: 319–321. 1577173 10.1016/0014-5793(92)80266-j

[20] Stadinski, B.D. , T. Delong , N. Reisdorph , *et al* 2010 Chromogranin A is an autoantigen in type 1 diabetes. Nat. Immunol. 11: 225–231. 20139986 10.1038/ni.1844 PMC3166626

[21] Jin, N. , Y. Wang , F. Crawford , *et al* 2015 N‐terminal additions to the WE14 peptide of chromogranin A create strong autoantigen agonists in type 1 diabetes. Proc. Natl. Acad. Sci. USA 112: 13318–13323. 26453556 10.1073/pnas.1517862112 PMC4629350

[22] Aardal, S. & K.B. Helle . 1992 The vasoinhibitory activity of bovine chromogranin A fragment (vasostatin) and its independence of extracellular calcium in isolated segments of human blood vessels. Regul. Pept. 41: 9–18. 1455014 10.1016/0167-0115(92)90509-s

[23] Aardal, S. , K.B. Helle , S. Elsayed , *et al* 1993 Vasostatins, comprising the N‐terminal domain of chromogranin A, suppress tension in isolated human blood vessel segments. J. Neuroendocrinol. 5: 405–412. 8401564 10.1111/j.1365-2826.1993.tb00501.x

[24] Tota, B. , R. Mazza , T. Angelone , *et al* 2003 Peptides from the N‐terminal domain of chromogranin A (vasostatins) exert negative inotropic effects in the isolated frog heart. Regul. Pept. 114: 91–99. 12832100 10.1016/s0167-0115(03)00112-5

[25] Corti, A. , C. Mannarino , R. Mazza , *et al* 2004 Chromogranin A N‐terminal fragments vasostatin‐1 and the synthetic CGA 7–57 peptide act as cardiostatins on the isolated working frog heart. Gen. Comp. Endocrinol. 136: 217–224. 15028525 10.1016/j.ygcen.2003.12.012

[26] Pike, S.E. , L. Yao , K.D. Jones , *et al* 1998 Vasostatin, a calreticulin fragment, inhibits angiogenesis and suppresses tumor growth. J. Exp. Med. 188: 2349–2356. 9858521 10.1084/jem.188.12.2349 PMC2212424

[27] Mahata, S.K. , D.T. O'Connor , M. Mahata , *et al* 1997 Novel autocrine feedback control of catecholamine release. A discrete chromogranin A fragment is a noncompetitive nicotinic cholinergic antagonist. J. Clin. Invest. 100: 1623–1633. 9294131 10.1172/JCI119686 PMC508344

[28] Mahata, S.K. , M. Mahata , R.J. Parmer , *et al* 1999 Desensitization of catecholamine release: the novel catecholamine release‐inhibitory peptide catestatin (chromogranin A_344–364_) acts at the receptor to prevent nicotinic cholinergic tolerance. J. Biol. Chem. 274: 2920–2928. 9915830 10.1074/jbc.274.5.2920

[29] Mahata, S.K. , M. Mahata , A.R. Wakade , *et al* 2000 Primary structure and function of the catecholamine release inhibitory peptide catestatin (chromogranin A344–364): identification of amino acid residues crucial for activity. Mol. Endocrinol. 14: 1525–1535. 11043569 10.1210/mend.14.10.0531

[30] Mahata, S.K. , N.R. Mahapatra , M. Mahata , *et al* 2003 Catecholamine secretory vesicle stimulus‐transcription coupling *in vivo*. Demonstration by a novel transgenic promoter/photoprotein reporter and inhibition of secretion and transcription by the chromogranin A fragment catestatin. J. Biol. Chem. 278: 32058–32067. 12799369 10.1074/jbc.M305545200

[31] Mahata, S.K. , M. Mahata , G. Wen , *et al* 2004 The catecholamine release‐inhibitory “catestatin” fragment of chromogranin a: naturally occurring human variants with different potencies for multiple chromaffin cell nicotinic cholinergic responses. Mol. Pharmacol. 66: 1180–1191. 15326220 10.1124/mol.104.002139

[32] Wen, G. , S.K. Mahata , P. Cadman , *et al* 2004 Both rare and common polymorphisms contribute functional variation at CHGA, a regulator of catecholamine physiology. Am. J. Hum. Genet. 74: 197–207. 14740315 10.1086/381399 PMC1181918

[33] Mahapatra, N.R. , D.T. O'Connor , S.M. Vaingankar , *et al* 2005 Hypertension from targeted ablation of chromogranin A can be rescued by the human ortholog. J. Clin. Invest. 115: 1942–1952. 16007257 10.1172/JCI24354 PMC1159140

[34] Briolat, J. , S.D. Wu , S.K. Mahata , *et al* 2005 New antimicrobial activity for the catecholamine release‐inhibitory peptide from chromogranin A. Cell. Mol. Life Sci. 62: 377–385. 15723172 10.1007/s00018-004-4461-9 PMC11924474

[35] Theurl, M. , W. Schgoer , K. Albrecht , *et al* 2010 The neuropeptide catestatin acts as a novel angiogenic cytokine via a basic fibroblast growth factor‐dependent mechanism. Circ. Res. 107: 1326–1335. 20930149 10.1161/CIRCRESAHA.110.219493 PMC10798356

[36] Angelone, T. , A.M. Quintieri , B.K. Brar , *et al* 2008 The antihypertensive chromogranin a peptide catestatin acts as a novel endocrine/paracrine modulator of cardiac inotropism and lusitropism. Endocrinology 149: 4780–4793. 18535098 10.1210/en.2008-0318 PMC2582908

[37] Radek, K.A. , B. Lopez‐Garcia , M. Hupe , *et al* 2008 The neuroendocrine peptide catestatin is a cutaneous antimicrobial and induced in the skin after injury. J. Invest. Dermatol. 128: 1525–1534. 18185531 10.1038/sj.jid.5701225 PMC2757066

[38] Zhang, D. , P. Shooshtarizadeh , B.J. Laventie , *et al* 2009 Two chromogranin a‐derived peptides induce calcium entry in human neutrophils by calmodulin‐regulated calcium independent phospholipase A2. PLoS One 4: e4501. 19225567 10.1371/journal.pone.0004501 PMC2639705

[39] Angelone, T. , A.M. Quintieri , T. Pasqua , *et al* 2012 Phosphodiesterase type‐2 and NO‐dependent S‐nitrosylation mediate the cardioinhibition of the antihypertensive catestatin. Am. J. Physiol. Heart Circ. Physiol. 302: H431–H442. 22058158 10.1152/ajpheart.00491.2011

[40] Bandyopadhyay, G.K. , C.U. Vu , S. Gentile , *et al* 2012 Catestatin (chromogranin A(352–372)) and novel effects on mobilization of fat from adipose tissue through regulation of adrenergic and leptin signaling. J. Biol. Chem. 287: 23141–23151. 22535963 10.1074/jbc.M111.335877 PMC3391131

[41] Crippa, L. , M. Bianco , B. Colombo , *et al* 2013 A new chromogranin A‐dependent angiogenic switch activated by thrombin. Blood 121: 392–402. 23190532 10.1182/blood-2012-05-430314 PMC3544118

[42] Koshimizu, H. , N.X. Cawley , T. Kim , *et al* 2011 Serpinin: a novel chromogranin A‐derived, secreted peptide up‐regulates protease nexin‐1 expression and granule biogenesis in endocrine cells. Mol. Endocrinol. 25: 732–744. 21436258 10.1210/me.2010-0124 PMC3082324

[43] Tota, B. , S. Gentile , T. Pasqua , *et al* 2012 The novel chromogranin A‐derived serpinin and pyroglutaminated serpinin peptides are positive cardiac β‐adrenergic‐like inotropes. FASEB J. 26: 2888–2898. 22459152 10.1096/fj.11-201111 PMC3382102

[44] Cerra, M.C. , L. De Iuri , T. Angelone , *et al* 2006 Recombinant N‐terminal fragments of chromogranin‐A modulate cardiac function of the Langendorff‐perfused rat heart. Basic Res. Cardiol. 101: 43–52. 16151967 10.1007/s00395-005-0547-2

[45] Gayen, J.R. , M. Saberi , S. Schenk , *et al* 2009 A novel pathway of insulin sensitivity in chromogranin A null mice: a crucial role for pancreastatin in glucose homeostasis. J. Biol. Chem. 284: 28498–28509. 19706599 10.1074/jbc.M109.020636 PMC2781393

[46] Gayen, J.R. , Y. Gu , D.T. O'Connor , *et al* 2009 Global disturbances in autonomic function yield cardiovascular instability and hypertension in the chromogranin A null mouse. Endocrinology 150: 5027–5035. 19819970 10.1210/en.2009-0429 PMC2775982

[47] Bandyopadhyay, G.K. , M. Lu , E. Avolio , *et al* 2015 Pancreastatin‐dependent inflammatory signaling mediates obesity‐induced insulin resistance. Diabetes 64: 104–116. 25048197 10.2337/db13-1747

[48] Pieroni, M. , A. Corti , B. Tota , *et al* 2007 Myocardial production of chromogranin A in human heart: a new regulatory peptide of cardiac function. Eur. Heart J. 28: 1117–1127. 17389654 10.1093/eurheartj/ehm022

[49] Estensen, M.E. , A. Hognestad , U. Syversen , *et al* 2006 Prognostic value of plasma chromogranin A levels in patients with complicated myocardial infarction. Am. Heart J. 152: 927.e921–926. 17070161 10.1016/j.ahj.2006.05.008

[50] Jansson, A.M. , H. Rosjo , T. Omland , *et al* 2009 Prognostic value of circulating chromogranin A levels in acute coronary syndromes. Eur. Heart J. 30: 25–32. 19028779 10.1093/eurheartj/ehn513 PMC2639087

[51] Manhenke, C. , S. Orn , S. von Haehling , *et al* 2013 Clustering of 37 circulating biomarkers by exploratory factor analysis in patients following complicated acute myocardial infarction. Int. J. Cardiol. 166: 729–735. 22197217 10.1016/j.ijcard.2011.11.089

[52] Ceconi, C. , R. Ferrari , T. Bachetti , *et al* 2002 Chromogranin A in heart failure; a novel neurohumoral factor and a predictor for mortality. Eur. Heart J. 23: 967–974. 12069452 10.1053/euhj.2001.2977

[53] Omland, T. , K. Dickstein & U. Syversen . 2003 Association between plasma chromogranin A concentration and long‐term mortality after myocardial infarction. Am. J. Med. 114: 25–30. 12543286 10.1016/s0002-9343(02)01425-0

[54] Rosjo, H. , S. Masson , R. Latini , *et al* 2010 Prognostic value of chromogranin A in chronic heart failure: data from the GISSI‐Heart Failure trial. Eur. J. Heart Fail. 12: 549–556. 20388648 10.1093/eurjhf/hfq055

[55] Corti, A. , F. Marcucci & T. Bachetti . 2018 Circulating chromogranin A and its fragments as diagnostic and prognostic disease markers. Pflugers Arch. 470: 199–210. 29018988 10.1007/s00424-017-2030-y

[56] Dieplinger, B. , A. Gegenhuber , J. Struck , *et al* 2009 Chromogranin A and C‐terminal endothelin‐1 precursor fragment add independent prognostic information to amino‐terminal proBNP in patients with acute destabilized heart failure. Clin. Chim. Acta 400: 91–96. 19000665 10.1016/j.cca.2008.10.012

[57] Goetze, J.P. , U. Alehagen , A. Flyvbjerg , *et al* 2014 Chromogranin A as a biomarker in cardiovascular disease. Biomark. Med. 8: 133–140. 24325234 10.2217/bmm.13.102

[58] Pan, W.Q. , Y.H. He , Q. Su , *et al* 2016 Association of decreased serum vasostatin‐2 level with ischemic chronic heart failure and with MACE in 3‐year follow‐up: vasostatin‐2 prevents heart failure in myocardial infarction rats. Int. J. Cardiol. 221: 1–11. 27395818 10.1016/j.ijcard.2016.06.065

[59] O'Connor, D.T. & K.N. Bernstein . 1984 Radioimmunoassay of chromogranin A in plasma as a measure of exocytotic sympathoadrenal activity in normal subjects and patients with pheochromocytoma. N. Engl. J. Med. 311: 764–770. 6472366 10.1056/NEJM198409203111204

[60] O'Connor, D.T. 1984 Chromogranin A: implications for hypertension. J. Hypertens. Suppl. 2: S147–S150. 6599663

[61] O'Connor, D.T. 1985 Plasma chromogranin A. Initial studies in human hypertension. Hypertension 7: I76–79. 3997234 10.1161/01.hyp.7.3_pt_2.i76

[62] Schober, M. , P.R. Howe , G. Sperk , *et al* 1989 An increased pool of secretory hormones and peptides in adrenal medulla of stroke‐prone spontaneously hypertensive rats. Hypertension 13: 469–474. 2566578 10.1161/01.hyp.13.5.469

[63] O'Connor, D.T. , M.A. Takiyyuddin , M.P. Printz , *et al* 1999 Catecholamine storage vesicle protein expression in genetic hypertension. Blood Press. 8: 285–295. 10803489 10.1080/080370599439508

[64] Inamdar, A.A. & A.C. Inamdar . 2016 Heart failure: diagnosis, management and utilization. J. Clin. Med. 5 10.3390/jcm5070062 PMC4961993 27367736

[65] Levy, D. , M.G. Larson , R.S. Vasan , *et al* 1996 The progression from hypertension to congestive heart failure. JAMA 275: 1557–1562. 8622246

[66] Messerli, F.H. , S.F. Rimoldi & S. Bangalore . 2017 The transition from hypertension to heart failure: contemporary update. JACC Heart Fail. 5: 543–551. 28711447 10.1016/j.jchf.2017.04.012

[67] Grassi, G. 2009 Assessment of sympathetic cardiovascular drive in human hypertension: achievements and perspectives. Hypertension 54: 690–697. 19720958 10.1161/HYPERTENSIONAHA.108.119883

[68] Parati, G. & M. Esler . 2012 The human sympathetic nervous system: its relevance in hypertension and heart failure. Eur. Heart J. 33: 1058–1066. 22507981 10.1093/eurheartj/ehs041

[69] Takiyyuddin, M.A. , M.R. Brown , T.Q. Dinh , *et al* 1994 Sympatho‐adrenal secretion in humans: factors governing catecholamine and storage vesicle peptide co‐release. J. Auton. Pharmacol. 14: 187–200. 7929473 10.1111/j.1474-8673.1994.tb00601.x

[70] Corti, A. , R. Ferrari & C. Ceconi . 2000 Chromogranin A and tumor necrosis factor‐alpha (TNF) in chronic heart failure. Adv. Exp. Med. Biol. 482: 351–359. 11192595 10.1007/0-306-46837-9_28

[71] Larsen, A.I. , K.B. Helle , M. Christensen , *et al* 2008 Effect of exercise training on chromogranin A and relationship to N‐ANP and inflammatory cytokines in patients with chronic heart failure. Int. J. Cardiol. 127: 117–120. 17586073 10.1016/j.ijcard.2007.04.012

[72] Tota, B. , T. Angelone & M.C. Cerra . 2014 The surging role of chromogranin A in cardiovascular homeostasis. Front. Chem. 2: 64. 25177680 10.3389/fchem.2014.00064 PMC4132265

[73] Angelone, T. , R. Mazza & M.C. Cerra . 2012 Chromogranin‐A: a multifaceted cardiovascular role in health and disease. Curr. Med. Chem. 19: 4042–4050. 22834795 10.2174/092986712802430009

[74] Helle, K.B. , A. Corti , M.H. Metz‐Boutigue & B. Tota . 2007 The endocrine role for chromogranin A: a prohormone for peptides with regulatory properties. Cell. Mol. Life Sci. 64: 2863–2886. 17717629 10.1007/s00018-007-7254-0 PMC11135995

[75] Angeletti, R.H. , S. Aardal , G. Serck‐Hanssen , *et al* 1994 Vasoinhibitory activity of synthetic peptides from the amino terminus of chromogranin A. Acta Physiol. Scand. 152: 11–19. 7810329 10.1111/j.1748-1716.1994.tb09780.x

[76] Troger, J. , M. Theurl , R. Kirchmair , *et al* 2017 Granin‐derived peptides. Prog. Neurobiol. 154: 37–61. 28442394 10.1016/j.pneurobio.2017.04.003

[77] Shooshtarizadeh, P. , D. Zhang , J.F. Chich , *et al* 2010 The antimicrobial peptides derived from chromogranin/secretogranin family, new actors of innate immunity. Regul. Pept. 165: 102–110. 19932135 10.1016/j.regpep.2009.11.014

[78] Rumio, C. , G.F. Dusio , B. Colombo , *et al* 2012 The N‐terminal fragment of chromogranin A, vasostatin‐1 protects mice from acute or chronic colitis upon oral administration. Dig. Dis. Sci. 57: 1227–1237. 22278339 10.1007/s10620-012-2031-9

[79] Salem, S. , V. Jankowski , Y. Asare , *et al* 2015 Identification of the vasoconstriction‐inhibiting factor (VIF), a potent endogenous cofactor of angiotensin II acting on the angiotensin II type 2 receptor. Circulation 131: 1426–1434. 25810338 10.1161/CIRCULATIONAHA.114.013168

[80] Cappello, S. , T. Angelone , B. Tota , *et al* 2007 Human recombinant chromogranin A‐derived vasostatin‐1 mimics preconditioning via an adenosine/nitric oxide signaling mechanism. Am. J. Physiol. Heart Circ. Physiol. 293: H719–H727. 17416598 10.1152/ajpheart.01352.2006

[81] Pasqua, T. , A. Corti , S. Gentile , *et al* 2013 Full‐length human chromogranin‐A cardioactivity: myocardial, coronary and stimulus‐induced processing evidence in normotensive and hypertensive male rat hearts. Endocrinology 154: 3353–3365. 23751870 10.1210/en.2012-2210

[82] Rocca, C. , F. Scavello , B. Colombo , *et al* 2019 Physiological levels of chromogranin A prevent doxorubicin‐induced cardiotoxicity without impairing its anticancer activity. FASEB J. 33: 7734–7747. 30973759 10.1096/fj.201802707R

[83] Kennedy, B.P. , S.K. Mahata , D.T. O'Connor , *et al* 1998 Mechanism of cardiovascular actions of the chromogranin A fragment catestatin *in vivo* . Peptides 19: 1241–1248. 9786174 10.1016/s0196-9781(98)00086-2

[84] Mahapatra, N.R. 2008 Catestatin is a novel endogenous peptide that regulates cardiac function and blood pressure. Cardiovasc. Res. 80: 330–338. 18541522 10.1093/cvr/cvn155

[85] Mahata, S.K. , M. Mahata , M.M. Fung , *et al* 2010 Catestatin: a multifunctional peptide from chromogranin A. Regul. Pept. 162: 33–43. 20116404 10.1016/j.regpep.2010.01.006 PMC2866790

[86] Fung, M.M. , R.M. Salem , P. Mehtani , *et al* 2010 Direct vasoactive effects of the chromogranin A (CHGA) peptide catestatin in humans *in vivo* . Clin. Exp. Hypertens. 32: 278–287. 20662728 10.3109/10641960903265246 PMC3109075

[87] Biswas, N. , J. Gayen , M. Mahata , *et al* 2012 Novel peptide isomer strategy for stable inhibition of catecholamine release: application to hypertension. Hypertension 60: 1552–1559. 23129699 10.1161/HYPERTENSIONAHA.112.202127 PMC3523723

[88] Dev, N.B. , J.R. Gayen , D.T. O'Connor , *et al* 2010 Chromogranin A and the autonomic system: decomposition of heart rate variability by time and frequency domains, along with non‐linear characteristics during chromogranin A ablation, with “rescue” by its catestatin. Endocrinology 151: 2760–2768. 20410203 10.1210/en.2009-1110 PMC2875835

[89] Mazza, R. , A. Gattuso , C. Mannarino , *et al* 2008 Catestatin (chromogranin A344–364) is a novel cardiosuppressive agent: inhibition of isoproterenol and endothelin signaling in the frog heart. Am. J. Physiol. Heart Circ. Physiol. 295: H113–H122. 18469147 10.1152/ajpheart.00172.2008 PMC2494752

[90] Imbrogno, S. , F. Garofalo , M.C. Cerra , *et al* 2010 The catecholamine release‐inhibitory peptide catestatin (chromogranin A344–364) modulates myocardial function in fish. J. Exp. Biol. 213: 3636–3643. 20952611 10.1242/jeb.045567

[91] Mahata, S.K. , M. Kiranmayi & N.R. Mahapatra . 2018 Catestatin: a master regulator of cardiovascular functions. Curr. Med. Chem. 25: 1352–1374. 28443506 10.2174/0929867324666170425100416

[92] Hill, S.J. 1992 Multiple histamine receptors: properties and functional characteristics. Biochem. Soc. Trans. 20: 122–125. 1321744 10.1042/bst0200122

[93] Kruger, P.G. , S.K. Mahata & K.B. Helle . 2003 Catestatin (CgA344–364) stimulates rat mast cell release of histamine in a manner comparable to mastoparan and other cationic charged neuropeptides. Regul. Pept. 114: 29–35. 12763637 10.1016/s0167-0115(03)00069-7

[94] Aung, G. , F. Niyonsaba , H. Ushio , *et al* 2011 Catestatin, a neuroendocrine antimicrobial peptide, induces human mast cell migration, degranulation and production of cytokines and chemokines. Immunology 132: 527–539. 21214543 10.1111/j.1365-2567.2010.03395.x PMC3075506

[95] Schafer, M.K. , S.K. Mahata , N. Stroth , *et al* 2010 Cellular distribution of chromogranin A in excitatory, inhibitory, aminergic and peptidergic neurons of the rodent central nervous system. Regul. Pept. 165: 36–44. 20005907 10.1016/j.regpep.2009.11.021 PMC2997928

[96] Mahata, S.K. , M. Mahata , J. Marksteiner , *et al* 1991 Distribution of mRNAs for chromogranins A and B and secretogranin II in rat brain. Eur. J. Neurosci. 3: 895–904. 12106456 10.1111/j.1460-9568.1991.tb00101.x

[97] Mahata, M. , S.K. Mahata , R. Fischer‐Colbrie , *et al* 1993 Ontogenic development and distribution of mRNAs of chromogranin A and B, secretogranin II, p65 and synaptin/synaptophysin in rat brain. Brain Res. Dev. Brain Res. 76: 43–58. 8306430 10.1016/0165-3806(93)90121-p

[98] Schafer, M.K. , D. Nohr , H. Romeo , *et al* 1994 Pan‐neuronal expression of chromogranin A in rat nervous system. Peptides 15: 263–279. 8008631 10.1016/0196-9781(94)90012-4

[99] Somogyi, P. , A.J. Hodgson , R.W. DePotter , *et al* 1984 Chromogranin immunoreactivity in the central nervous system. Immunochemical characterisation, distribution and relationship to catecholamine and enkephalin pathways. Brain Res. 320: 193–230. 6084534 10.1016/0165-0173(84)90007-9

[100] Guyenet, P.G. 2006 The sympathetic control of blood pressure. Nat. Rev. Neurosci. 7: 335–346. 16760914 10.1038/nrn1902

[101] Gaede, A.H. & P.M. Pilowsky . 2012 Catestatin, a chromogranin A‐derived peptide, is sympathoinhibitory and attenuates sympathetic barosensitivity and the chemoreflex in rat CVLM. Am. J. Physiol. Regul. Integr. Comp. Physiol. 302: R365–R372. 22129620 10.1152/ajpregu.00409.2011

[102] Avolio, E. , S.K. Mahata , E. Mantuano , *et al* 2014 Antihypertensive and neuroprotective effects of catestatin in spontaneously hypertensive rats: interaction with GABAergic transmission in amygdala and brainstem. Neuroscience 270: 48–57. 24731867 10.1016/j.neuroscience.2014.04.001 PMC10843893

[103] Bassino, E. , S. Fornero , M.P. Gallo , *et al* 2011 A novel catestatin‐induced antiadrenergic mechanism triggered by the endothelial PI3K‐eNOS pathway in the myocardium. Cardiovasc. Res. 91: 617–624. 21543385 10.1093/cvr/cvr129 PMC3156905

[104] Angelone, T. , A.M. Quintieri , T. Pasqua , *et al* 2015 The NO stimulator, Catestatin, improves the Frank–Starling response in normotensive and hypertensive rat hearts. Nitric Oxide 50: 10–19. 26241941 10.1016/j.niox.2015.07.004

[105] Penna, C. , G. Alloatti , M.P. Gallo , *et al* 2010 Catestatin improves post‐ischemic left ventricular function and decreases ischemia/reperfusion injury in heart. Cell. Mol. Neurobiol. 30: 1171–1179. 21104119 10.1007/s10571-010-9598-5 PMC3008938

[106] Perrelli, M.G. , F. Tullio , C. Angotti , *et al* 2013 Catestatin reduces myocardial ischaemia/reperfusion injury: involvement of PI3K/Akt, PKCs, mitochondrial K(ATP) channels and ROS signalling. Pflugers Arch. 465: 1031–1040. 23319164 10.1007/s00424-013-1217-0

[107] Bassino, E. , S. Fornero , M.P. Gallo , *et al* 2015 Catestatin exerts direct protective effects on rat cardiomyocytes undergoing ischemia/reperfusion by stimulating PI3K‐Akt‐GSK3β pathway and preserving mitochondrial membrane potential. PLoS One 10: e0119790. 25774921 10.1371/journal.pone.0119790 PMC4361546

[108] Sahu, B.S. , J.M. Obbineni , G. Sahu , *et al* 2012 Functional genetic variants of the catecholamine‐release‐inhibitory peptide catestatin in an Indian population: allele‐specific effects on metabolic traits. J. Biol. Chem. 287: 43840–43852. 23105094 10.1074/jbc.M112.407916 PMC3527967

[109] Kiranmayi, M. , V.R. Chirasani , P.K. Allu , *et al* 2016 Catestatin Gly364Ser variant alters systemic blood pressure and the risk for hypertension in human populations via endothelial nitric oxide pathway. Hypertension 68: 334–347. 27324226 10.1161/HYPERTENSIONAHA.116.06568 PMC4945419

[110] Rao, F. , G. Wen , J.R. Gayen , *et al* 2007 Catecholamine release‐inhibitory peptide catestatin (chromogranin A(352–372)): naturally occurring amino acid variant Gly364Ser causes profound changes in human autonomic activity and alters risk for hypertension. Circulation 115: 2271–2281. 17438154 10.1161/CIRCULATIONAHA.106.628859

[111] Pasqua, T. , B. Tota , C. Penna , *et al* 2015 pGlu‐serpinin protects the normotensive and hypertensive heart from ischemic injury. J. Endocrinol. 227: 167–178. 26400960 10.1530/JOE-15-0199 PMC4651656

[112] Ying, W. , S. Mahata , G.K. Bandyopadhyay , *et al* 2018 Catestatin inhibits obesity‐induced macrophage infiltration and inflammation in the liver and suppresses hepatic glucose production, leading to improved insulin sensitivity. Diabetes 67: 841–848. 29432123 10.2337/db17-0788 PMC6463753

[113] Mathis, D. & S.E. Shoelson . 2011 Immunometabolism: an emerging frontier. Nat. Rev. Immunol. 11: 81. 21469396 10.1038/nri2922 PMC4784680

[114] Weisberg, S.P. , D. McCann , M. Desai , *et al* 2003 Obesity is associated with macrophage accumulation in adipose tissue. J. Clin. Invest. 112: 1796–1808. 14679176 10.1172/JCI19246 PMC296995

[115] Xu, H. , G.T. Barnes , Q. Yang , *et al* 2003 Chronic inflammation in fat plays a crucial role in the development of obesity‐related insulin resistance. J. Clin. Invest. 112: 1821–1830. 14679177 10.1172/JCI19451 PMC296998

[116] Lumeng, C.N. , J.L. Bodzin & A.R. Saltiel . 2007 Obesity induces a phenotypic switch in adipose tissue macrophage polarization. J. Clin. Invest. 117: 175–184. 17200717 10.1172/JCI29881 PMC1716210

[117] Wu, D. , A.B. Molofsky , H.E. Liang , *et al* 2011 Eosinophils sustain adipose alternatively activated macrophages associated with glucose homeostasis. Science 332: 243–247. 21436399 10.1126/science.1201475 PMC3144160

[118] Talukdar, S. , Y. Oh da , G. Bandyopadhyay , *et al* 2012 Neutrophils mediate insulin resistance in mice fed a high‐fat diet through secreted elastase. Nat. Med. 18: 1407–1412. 22863787 10.1038/nm.2885 PMC3491143

[119] Molofsky, A.B. , J.C. Nussbaum , H.E. Liang , *et al* 2013 Innate lymphoid type 2 cells sustain visceral adipose tissue eosinophils and alternatively activated macrophages. J. Exp. Med. 210: 535–549. 23420878 10.1084/jem.20121964 PMC3600903

[120] Nussbaum, J.C. , S.J. Van Dyken , J. von Moltke , *et al* 2013 Type 2 innate lymphoid cells control eosinophil homeostasis. Nature 502: 245–248. 24037376 10.1038/nature12526 PMC3795960

[121] Lee, B.C. , M.S. Kim , M. Pae , *et al* 2016 Adipose natural killer cells regulate adipose tissue macrophages to promote insulin resistance in obesity. Cell Metab. 23: 685–698. 27050305 10.1016/j.cmet.2016.03.002 PMC4833527

[122] Boulenouar, S. , X. Michelet , D. Duquette , *et al* 2017 Adipose type one innate lymphoid cells regulate macrophage homeostasis through targeted cytotoxicity. Immunity 46: 273–286. 28228283 10.1016/j.immuni.2017.01.008

[123] Theurich, S. , E. Tsaousidou , R. Hanssen , *et al* 2017 IL‐6/stat3‐dependent induction of a distinct, obesity‐associated NK cell subpopulation deteriorates energy and glucose homeostasis. Cell Metab. 26: 171–184.e176. 28683285 10.1016/j.cmet.2017.05.018

[124] Muntjewerff, E.M. , G. Dunkel , M.J.T. Nicolasen , *et al* 2018 Catestatin as a target for treatment of inflammatory diseases. Front. Immunol. 9: 2199. 30337922 10.3389/fimmu.2018.02199 PMC6180191

[125] Nishimura, S. , I. Manabe , M. Nagasaki , *et al* 2009 CD8^+^ effector T cells contribute to macrophage recruitment and adipose tissue inflammation in obesity. Nat. Med. 15: 914–920. 19633658 10.1038/nm.1964

[126] Winer, S. , Y. Chan , G. Paltser , *et al* 2009 Normalization of obesity‐associated insulin resistance through immunotherapy. Nat. Med. 15: 921–929. 19633657 10.1038/nm.2001 PMC3063199

[127] Feuerer, M. , L. Herrero , D. Cipolletta , *et al* 2009 Lean, but not obese, fat is enriched for a unique population of regulatory T cells that affect metabolic parameters. Nat. Med. 15: 930–939. 19633656 10.1038/nm.2002 PMC3115752

[128] Winer, D.A. , S. Winer , L. Shen , *et al* 2011 B cells promote insulin resistance through modulation of T cells and production of pathogenic IgG antibodies. Nat. Med. 17: 610–617. 21499269 10.1038/nm.2353 PMC3270885

[129] Cipolletta, D. , M. Feuerer , A. Li , *et al* 2012 PPAR‐γ is a major driver of the accumulation and phenotype of adipose tissue Treg cells. Nature 486: 549–553. 22722857 10.1038/nature11132 PMC3387339

[130] Huh, J.Y. , J.I. Kim , Y.J. Park , *et al* 2013 A novel function of adipocytes in lipid antigen presentation to iNKT cells. Mol. Cell. Biol. 33: 328–339. 23149942 10.1128/MCB.00552-12 PMC3554106

[131] Lynch, L. 2014 Adipose invariant natural killer T cells. Immunology 142: 337–346. 24673647 10.1111/imm.12269 PMC4080949

[132] Ying, W. , J. Wollam , J.M. Ofrecio , *et al* 2017 Adipose tissue B2 cells promote insulin resistance through leukotriene LTB4/LTB4R1 signaling. J. Clin. Invest. 127: 1019–1030. 28192375 10.1172/JCI90350 PMC5330737

[133] Hotamisligil, G.S. , N.S. Shargill & B.M. Spiegelman . 1993 Adipose expression of tumor necrosis factor‐alpha: direct role in obesity‐linked insulin resistance. Science 259: 87–91. 7678183 10.1126/science.7678183

[134] Li, P. , D.Y. Oh , G. Bandyopadhyay , *et al* 2015 LTB4 promotes insulin resistance in obese mice by acting on macrophages, hepatocytes and myocytes. Nat. Med. 21: 239–247. 25706874 10.1038/nm.3800 PMC4429798

[135] Li, P. , S. Liu , M. Lu , *et al* 2016 Hematopoietic‐derived galectin‐3 causes cellular and systemic insulin resistance. Cell 167: 973–984.e912. 27814523 10.1016/j.cell.2016.10.025 PMC5179329

[136] Chavez, J.A. & S.A. Summers . 2012 A ceramide‐centric view of insulin resistance. Cell Metab. 15: 585–594. 22560211 10.1016/j.cmet.2012.04.002

[137] Holland, W.L. , B.T. Bikman , L.P. Wang , *et al* 2011 Lipid‐induced insulin resistance mediated by the proinflammatory receptor TLR4 requires saturated fatty acid‐induced ceramide biosynthesis in mice. J. Clin. Invest. 121: 1858–1870. 21490391 10.1172/JCI43378 PMC3083776

[138] Cai, D. , M. Yuan , D.F. Frantz , *et al* 2005 Local and systemic insulin resistance resulting from hepatic activation of IKK‐beta and NF‐kappaB. Nat. Med. 11: 183–190. 15685173 10.1038/nm1166 PMC1440292

[139] Sabio, G. , J. Cavanagh‐Kyros , H.J. Ko , *et al* 2009 Prevention of steatosis by hepatic JNK1. Cell Metab. 10: 491–498. 19945406 10.1016/j.cmet.2009.09.007 PMC2804105

[140] Kojima, M. , N. Ozawa , Y. Mori , *et al* 2018 Catestatin prevents macrophage‐driven atherosclerosis but not arterial injury‐induced neointimal hyperplasia. Thromb. Haemost. 118: 182–194. 29304538 10.1160/TH17-05-0349

[141] Ahren, B. , G. Bertrand , M. Roye , *et al* 1996 Pancreastatin modulates glucose‐stimulated insulin secretion from the perfused rat pancreas. Acta Physiol. Scand. 158: 63–70. 8876749 10.1046/j.1365-201X.1996.525291000.x

[142] Efendic, S. , K. Tatemoto , V. Mutt , *et al* 1987 Pancreastatin and islet hormone release. Proc. Natl. Acad. Sci. USA 84: 7257–7260. 2890162 10.1073/pnas.84.20.7257 PMC299271

[143] Peiro, E. , P. Miralles , R.A. Silvestre , *et al* 1989 Pancreastatin inhibits insulin secretion as induced by glucagon, vasoactive intestinal peptide, gastric inhibitory peptide, and 8‐cholecystokinin in the perfused rat pancreas. Metabolism 38: 679–682. 2661967 10.1016/0026-0495(89)90107-8

[144] Peiro, E. , P. Degano , R.A. Silvestre , *et al* 1991 Inhibition of insulin release by amylin is not mediated by changes in somatostatin output. Life Sci. 49: 761–765. 1678849 10.1016/0024-3205(91)90109-o

[145] Schmidt, W.E. & W. Creutzfeldt . 1991 Pancreastatin–a novel regulatory peptide? Acta Oncol. 30: 441–449. 1854501 10.3109/02841869109092399

[146] von Schonfeld, J. , J. Kleimann , M.K. Muller , *et al* 1991 Glucose‐dependent effects of pancreastatin on insulin and glucagon release. Int. J. Pancreatol. 10: 143–149. 1748828 10.1007/BF02924117

[147] Peiro, E. , P. Degano , P. Miralles , *et al* 1991 Homologous pancreastatin inhibits insulin secretion without affecting glucagon and somatostatin release in the perfused rat pancreas. Regul. Pept. 34: 159–167. 1681569 10.1016/0167-0115(91)90175-g

[148] Gonzalez‐Yanes, C. & V. Sánchez‐Margalet . 2000 Pancreastatin modulates insulin signaling in rat adipocytes: mechanisms of cross‐talk. Diabetes 49: 1288–1294. 10923627 10.2337/diabetes.49.8.1288

[149] O'Connor, D.T. , P.E. Cadman , C. Smiley , *et al* 2005 Pancreastatin: multiple actions on human intermediary metabolism *in vivo*, variation in disease, and naturally occurring functional genetic polymorphism. J. Clin. Endocrinol. Metab. 90: 5414–5425. 15956083 10.1210/jc.2005-0408

[150] Biswas, N. , R.S. Friese , J.R. Gayen , *et al* 2014 Discovery of a novel target for the dysglycemic chromogranin A fragment pancreastatin: interaction with the chaperone GRP78 to influence metabolism. PLoS One 9: e84132. 24465394 10.1371/journal.pone.0084132 PMC3896336

[151] Allu, P.K. , V.R. Chirasani , D. Ghosh , *et al* 2014 Naturally occurring variants of the dysglycemic peptide pancreastatin: differential potencies for multiple cellular functions and structure–function correlation. J. Biol. Chem. 289: 4455–4469. 24338022 10.1074/jbc.M113.520916 PMC3924307

[152] Bandyopadhyay, G.K. & S.K. Mahata . 2017 Chromogranin A regulation of obesity and peripheral insulin sensitivity. Front. Endocrinol. (Lausanne) 8: 20. 28228748 10.3389/fendo.2017.00020 PMC5296320

[153] Sanchez, V. , M. Lucas , J.R. Calvo , *et al* 1992 Glycogenolytic effect of pancreastatin in isolated rat hepatocytes is mediated by a cyclic‐AMP‐independent Ca(2^+^)‐dependent mechanism. Biochem. J. 284: 659–662. 1377910 10.1042/bj2840659 PMC1132588

[154] Sanchez, V. , J.R. Calvo & R. Goberna . 1990 Glycogenolytic effect of pancreastatin in the rat. Biosci. Rep. 10: 87–91. 2187544 10.1007/BF01116856

[155] Sanchez‐Margalet, V. , J.R. Calvo & R. Goberna . 1992 Glucogenolytic and hyperglycemic effect of 33–49 C‐terminal fragment of pancreastatin in the rat *in vivo* . Horm. Metab. Res. 24: 455–457. 1464408 10.1055/s-2007-1003361

[156] Sanchez‐Margalet, V. & C. Gonzalez‐Yanes . 1998 Pancreastatin inhibits insulin action in rat adipocytes. Am. J. Physiol. 275: E1055–E1060. 9843749 10.1152/ajpendo.1998.275.6.E1055

[157] Yudkin, J.S. , C.D. Stehouwer , J.J. Emeis , *et al* 1999 C‐reactive protein in healthy subjects: associations with obesity, insulin resistance, and endothelial dysfunction: a potential role for cytokines originating from adipose tissue? Arterioscler. Thromb. Vasc. Biol. 19: 972–978. 10195925 10.1161/01.atv.19.4.972

[158] Festa, A. , R. D'Agostino & G. Howard, Jr. , *et al* 2000 Chronic subclinical inflammation as part of the insulin resistance syndrome: the Insulin Resistance Atherosclerosis Study (IRAS). Circulation 102: 42–47. 10880413 10.1161/01.cir.102.1.42

[159] Pradhan, A.D. , J.E. Manson , N. Rifai , *et al* 2001 C‐reactive protein, interleukin 6, and risk of developing type 2 diabetes mellitus. JAMA 286: 327–334. 11466099 10.1001/jama.286.3.327

[160] Glass, C.K. & J.M. Olefsky . 2012 Inflammation and lipid signaling in the etiology of insulin resistance. Cell Metab. 15: 635–645. 22560216 10.1016/j.cmet.2012.04.001 PMC4156155

[161] Ray, I. , S.K. Mahata & R.K. De . 2016 Obesity: an immunometabolic perspective. Front. Endocrinol. (Lausanne) 7: 157. 28018292 10.3389/fendo.2016.00157 PMC5149556

[162] Pini, M. , D.H. Rhodes , K.J. Castellanos , *et al* 2012 Rosiglitazone improves survival and hastens recovery from pancreatic inflammation in obese mice. PLoS One 7: e40944. 22815875 10.1371/journal.pone.0040944 PMC3397967

[163] Zhao, D. , B.H. McCully & V.L. Brooks . 2012 Rosiglitazone improves insulin sensitivity and baroreflex gain in rats with diet‐induced obesity. J. Pharmacol. Exp. Therap. 343: 206–213. 22815534 10.1124/jpet.112.194738 PMC3464031

[164] Foryst‐Ludwig, A. , M. Hartge , M. Clemenz , *et al* 2010 PPARgamma activation attenuates T‐lymphocyte‐dependent inflammation of adipose tissue and development of insulin resistance in obese mice. Cardiovasc. Diabetol. 9: 64. 20955583 10.1186/1475-2840-9-64 PMC2984486

[165] Helle, K.B. & A. Corti . 2015 Chromogranin A: a paradoxical player in angiogenesis and vascular biology. Cell. Mol. Life Sci. 72: 339–348. 25297920 10.1007/s00018-014-1750-9 PMC11113878

[166] Gregorc, V. , A. Spreafico , I. Floriani , *et al* 2007 Prognostic value of circulating chromogranin A and soluble tumor necrosis factor receptors in advanced nonsmall cell lung cancer. Cancer 110: 845–853. 17599769 10.1002/cncr.22856

[167] Corti, A. 2010 Chromogranin A and the tumor microenvironment. Cell. Mol. Neurobiol. 30: 1163–1170. 21080056 10.1007/s10571-010-9587-8 PMC11498746

[168] Giusti, M. , M. Sidoti , C. Augeri , *et al* 2004 Effect of short‐term treatment with low dosages of the proton‐pump inhibitor omeprazole on serum chromogranin A levels in man. Eur. J. Endocrinol. 150: 299–303. 15012614 10.1530/eje.0.1500299

[169] Sanduleanu, S. , M. Stridsberg , D. Jonkers , *et al* 1999 Serum gastrin and chromogranin A during medium‐ and long‐term acid suppressive therapy: a case–control study. Aliment. Pharmacol. Ther. 13: 145–153. 10102943 10.1046/j.1365-2036.1999.00466.x

[170] Bianco, M. , A. Gasparri , L. Generoso , *et al* 2016 Inhibition of chronic lymphocytic leukemia progression by full‐length chromogranin A and its N‐terminal fragment in mouse models. Oncotarget 7: 41725–41736. 27203389 10.18632/oncotarget.9407 PMC5173091

[171] Folkman, J. 2007 Angiogenesis: an organizing principle for drug discovery? Nat. Rev. Drug Discov. 6: 273–286. 17396134 10.1038/nrd2115

[172] Italiano, J.E., Jr. , J.L. Richardson , S. Patel‐Hett , *et al* 2008 Angiogenesis is regulated by a novel mechanism: pro‐ and antiangiogenic proteins are organized into separate platelet alpha granules and differentially released. Blood 111: 1227–1233. 17962514 10.1182/blood-2007-09-113837 PMC2214735

[173] Ribatti, D. 2009 Endogenous inhibitors of angiogenesis: a historical review. Leuk. Res. 33: 638–644. 19117606 10.1016/j.leukres.2008.11.019

[174] Belloni, D. , S. Scabini , C. Foglieni , *et al* 2007 The vasostatin‐I fragment of chromogranin A inhibits VEGF‐induced endothelial cell proliferation and migration. FASEB J. 21: 3052–3062. 17566084 10.1096/fj.06-6829com

[175] Veschini, L. , L. Crippa , E. Dondossola , *et al* 2011 The vasostatin‐1 fragment of chromogranin A preserves a quiescent phenotype in hypoxia‐driven endothelial cells and regulates tumor neovascularization. FASEB J. 25: 3906–3914. 21825034 10.1096/fj.11-182410

[176] Ferrero, E. , S. Scabini , E. Magni , *et al* 2004 Chromogranin A protects vessels against tumor necrosis factor alpha‐induced vascular leakage. FASEB J. 18: 554–556. 14734634 10.1096/fj.03-0922fje

[177] Dondossola, E. , L. Crippa , B. Colombo , *et al* 2012 Chromogranin A regulates tumor self‐seeding and dissemination. Cancer Res. 72: 449–459. 22139379 10.1158/0008-5472.CAN-11-2944

[178] Curnis, F. , A. Dallatomasina , M. Bianco , *et al* 2016 Regulation of tumor growth by circulating full‐length chromogranin A. Oncotarget 7: 72716–72732. 27683038 10.18632/oncotarget.12237 PMC5341939

[179] Selbonne, S. , F. Azibani , S. Iatmanen , *et al* 2012 *In vitro* and *in vivo* antiangiogenic properties of the serpin protease nexin‐1. Mol. Cell. Biol. 32: 1496–1505. 22331468 10.1128/MCB.06554-11 PMC3318585

[180] McKee, C.M. , D. Xu , Y. Cao , *et al* 2012 Protease nexin 1 inhibits hedgehog signaling in prostate adenocarcinoma. J. Clin. Invest. 122: 4025–4036. 23041623 10.1172/JCI59348 PMC3484431

[181] Bouton, M.C. , Y. Boulaftali , B. Richard , *et al* 2012 Emerging role of serpinE2/protease nexin‐1 in hemostasis and vascular biology. Blood 119: 2452–2457. 22234688 10.1182/blood-2011-10-387464

[182] Maestroni, S. , A. Maestroni , S. Ceglia , *et al* 2015 Effect of chromogranin A‐derived vasostatin‐1 on laser‐induced choroidal neovascularization in the mouse. Acta Ophthalmol. 93: e218–e22. 25271003 10.1111/aos.12557

[183] Bianco, M. , A.M. Gasparri , B. Colombo , *et al* 2016 Chromogranin A is preferentially cleaved into pro‐angiogenic peptides in the bone marrow of multiple myeloma patients. Cancer Res. 76: 1781–1791. 26869462 10.1158/0008-5472.CAN-15-1637

[184] Dallatomasina, A. , A.M. Gasparri , B. Colombo , *et al* 2019 Spatiotemporal regulation of tumor angiogenesis by circulating chromogranin A cleavage and neuropilin‐1 engagement. Cancer Res. 79: 1925–1937. 30796053 10.1158/0008-5472.CAN-18-0289

[185] Teesalu, T. , K.N. Sugahara , V.R. Kotamraju , *et al* 2009 C‐end rule peptides mediate neuropilin‐1‐dependent cell, vascular, and tissue penetration. Proc. Natl. Acad. Sci. USA 106: 16157–16162. 19805273 10.1073/pnas.0908201106 PMC2752543

[186] Teesalu, T. , K.N. Sugahara & E. Ruoslahti . 2013 Tumor‐penetrating peptides. Front. Oncol. 3: 216. 23986882 10.3389/fonc.2013.00216 PMC3753659

[187] Taupenot, L. , S.K. Mahata , M. Mahata , *et al* 2000 Interaction of the catecholamine release‐inhibitory peptide catestatin (chromogranin A_352–372_) with the chromaffin cell surface and Torpedo electroplax: implications for nicotinic cholinergic antagonism. Regul. Pept. 95: 9–17. 11062327 10.1016/s0167-0115(00)00135-x

[188] Sahu, B.S. , J. Mohan , G. Sahu , *et al* 2012 Molecular interactions of the physiological anti‐hypertensive peptide catestatin with the neuronal nicotinic acetylcholine receptor. J. Cell Sci. 125: 2323–2337. 22357947 10.1242/jcs.103176

[189] Pena, V.B. , I.C. Bonini , S.S. Antollini , *et al* 2011 Alpha 7‐type acetylcholine receptor localization and its modulation by nicotine and cholesterol in vascular endothelial cells. J. Cell. Biochem. 112: 3276–3288. 21748784 10.1002/jcb.23254

[190] Cooke, J.P. & Y.T. Ghebremariam . 2008 Endothelial nicotinic acetylcholine receptors and angiogenesis. Trends Cardiovasc. Med. 18: 247–253. 19232953 10.1016/j.tcm.2008.11.007 PMC2673464

[191] Arias, H.R. , V.E. Richards , D. Ng , *et al* 2009 Role of non‐neuronal nicotinic acetylcholine receptors in angiogenesis. Int. J. Biochem. Cell Biol. 41: 1441–1451. 19401144 10.1016/j.biocel.2009.01.013

[192] Heeschen, C. , J.J. Jang , M. Weis , *et al* 2001 Nicotine stimulates angiogenesis and promotes tumor growth and atherosclerosis. Nat. Med. 7: 833–839. 11433349 10.1038/89961

[193] Chiorazzi, N. , K.R. Rai & M. Ferrarini . 2005 Chronic lymphocytic leukemia. N. Engl. J. Med. 352: 804–815. 15728813 10.1056/NEJMra041720

[194] Minchinton, A.I. & I.F. Tannock . 2006 Drug penetration in solid tumours. Nat. Rev. Cancer 6: 583–592. 16862189 10.1038/nrc1893

[195] Jain, R.K. 1994 Barriers to drug delivery in solid tumors. Sci. Am. 271: 58–65. 10.1038/scientificamerican0794-58 8066425

[196] Tredan, O. , C.M. Galmarini , K. Patel , *et al* 2007 Drug resistance and the solid tumor microenvironment. J. Natl. Cancer Inst. 99: 1441–1454. 17895480 10.1093/jnci/djm135

[197] Curnis, F. , A. Sacchi , L. Borgna , *et al* 2000 Enhancement of tumor necrosis factor alpha antitumor immunotherapeutic properties by targeted delivery to aminopeptidase N (CD13). Nat. Biotechnol. 18: 1185–1190. 11062439 10.1038/81183

[198] Curnis, F. , A. Sacchi & A. Corti . 2002 Improving chemotherapeutic drug penetration in tumors by vascular targeting and barrier alteration. J. Clin. Invest. 110: 475–482. 12189241 10.1172/JCI15223 PMC150417

[199] Corti, A. , F. Curnis , G. Rossoni , *et al* 2013 Peptide‐mediated targeting of cytokines to tumor vasculature: the NGR‐hTNF example. BioDrugs 27: 591–603. 23743670 10.1007/s40259-013-0048-z PMC3832761

[200] Sacchi, A. , A. Gasparri , C. Gallo‐Stampino , *et al* 2006 Synergistic antitumor activity of cisplatin, paclitaxel, and gemcitabine with tumor vasculature‐targeted tumor necrosis factor‐alpha. Clin. Cancer Res. 12: 175–182. 16397040 10.1158/1078-0432.CCR-05-1147

[201] Ferreri, A.J.M. , T. Calimeri , G.M. Conte , *et al* 2019 R‐CHOP preceded by blood–brain barrier permeabilization with engineered tumor necrosis factor‐α in primary CNS lymphoma. Blood 134: 252–262. 31118164 10.1182/blood.2019000633

[202] Blois, A. , H. Holmsen , G. Martino , *et al* 2006 Interactions of chromogranin A‐derived vasostatins and monolayers of phosphatidylserine, phosphatidylcholine and phosphatidylethanolamine. Regul. Pept. 134: 30–37. 16445995 10.1016/j.regpep.2005.11.003

[203] Di Comite, G. , C.M. Rossi , A. Marinosci , *et al* 2009 Circulating chromogranin A reveals extra‐articular involvement in patients with rheumatoid arthritis and curbs TNF‐alpha‐elicited endothelial activation. J. Leukoc. Biol. 85: 81–87. 18832606 10.1189/jlb.0608358

[204] Curnis, F. , A.M. Gasparri , R. Longhi , *et al* 2012 Chromogranin A binds to αvβ6‐integrin and promotes wound healing in mice. Cell. Mol. Life Sci. 69: 2791–2803. 22415324 10.1007/s00018-012-0955-z PMC11114517

[205] Tsigelny, I. , S.K. Mahata , L. Taupenot , *et al* 1998 Mechanism of action of chromogranin A on catecholamine release: molecular modeling of the catestatin region reveals a beta‐strand/loop/beta‐strand structure secured by hydrophobic interactions and predictive of activity. Regul. Pept. 77: 43–53. 9809795 10.1016/s0167-0115(98)00040-8 PMC3676947

[206] Herrero, C.J. , E. Ales , A.J. Pintado , *et al* 2002 Modulatory mechanism of the endogenous peptide catestatin on neuronal nicotinic acetylcholine receptors and exocytosis. J. Neurosci. 22: 377–388. 11784782 10.1523/JNEUROSCI.22-02-00377.2002 PMC6758662

[207] Cryer, P.E. , J. Wortsman , S.D. Shah , *et al* 1991 Plasma chromogranin A as a marker of sympathochromaffin activity in humans. Am. J. Physiol. 260: E243–E246. 1996627 10.1152/ajpendo.1991.260.2.E243

[208] Tombetti, E. , B. Colombo , M.C. Di Chio , *et al* 2016 Chromogranin‐A production and fragmentation in patients with Takayasu arteritis. Arthritis Res. Ther. 18: 187. 27531191 10.1186/s13075-016-1082-2 PMC4987982

[209] O'Connor, D.T. , M.R. Pandlan , E. Carlton , *et al* 1989 Rapid radioimmunoassay of circulating chromogranin A: *in vitro* stability, exploration of the neuroendocrine character of neoplasia, and assessment of the effects of organ failure. Clin. Chem. 35: 1631–1637. 2547534

[210] Spadaro, A. , A. Ajello , C. Morace , *et al* 2005 Serum chromogranin‐A in hepatocellular carcinoma: diagnostic utility and limits. World J. Gastroenterol. 11: 1987–1990. 15800991 10.3748/wjg.v11.i13.1987 PMC4305722

[211] Sobol, R.E. , D.T. O'Connor , J. Addison , *et al* 1986 Elevated serum chromogranin A concentrations in small‐cell lung carcinoma. Ann. Intern. Med. 105: 698–700. 3021037 10.7326/0003-4819-105-5-698

[212] Hsu, C.H. , L.F. Reyes , C.J. Orihuela , *et al* 2015 Chromogranin A levels and mortality in patients with severe sepsis. Biomarkers 20: 171–176. 26154393 10.3109/1354750X.2015.1046932 PMC5027645

[213] Zhang, D. , T. Lavaux , A.C. Voegeli , *et al* 2008 Prognostic value of chromogranin A at admission in critically ill patients: a cohort study in a medical intensive care unit. Clin. Chem. 54: 1497–1503. 18635750 10.1373/clinchem.2007.102442

[214] Rosjo, H. , S. Nygard , K.M. Kaukonen , *et al* 2012 Prognostic value of chromogranin A in severe sepsis: data from the FINNSEPSIS study. Intensive Care Med. 38: 820–829. 22491939 10.1007/s00134-012-2546-8

[215] Teggi, R. , B. Colombo , M. Trimarchi , *et al* 2015 Altered chromogranin A circulating levels in Meniere's disease. Dis. Markers 2015: 643420. 25983374 10.1155/2015/643420 PMC4423029

[216] Ferrero, E. , A. Corti , J. Haroche , *et al* 2016 Plasma chromogranin A as a marker of cardiovascular involvement in Erdheim–Chester disease. Oncoimmunology 5: e1181244. 27622037 10.1080/2162402X.2016.1181244 PMC5006912

[217] O'Connor, D.T. & L.J. Deftos . 1986 Secretion of chromogranin A by peptide‐producing endocrine neoplasms. N. Engl. J. Med. 314: 1145–1151. 3007986 10.1056/NEJM198605013141803

[218] Hsiao, R.J. , H.P. Neumann , R.J. Parmer , *et al* 1990 Chromogranin A in familial pheochromocytoma: diagnostic screening value, prediction of tumor mass, and post‐resection kinetics indicating two‐compartment distribution. Am. J. Med. 88: 607–613. 2189303 10.1016/0002-9343(90)90526-j

[219] Blind, E. , H. Schmidt‐Gayk , H.P. Sinn , *et al* 1992 Chromogranin A as tumor marker in medullary thyroid carcinoma. Thyroid 2: 5–10. 1356053 10.1089/thy.1992.2.5

[220] Lee, S.H. , J.H. Jo , Y.J. Kim , *et al* 2019 Plasma chromogranin A as a prognostic marker in pancreatic ductal adenocarcinoma. Pancreas 48: 662–669. 31091213 10.1097/MPA.0000000000001319

